# HIF1A-dependent induction of alveolar epithelial PFKFB3 dampens acute lung injury

**DOI:** 10.1172/jci.insight.157855

**Published:** 2022-12-22

**Authors:** Christine U. Vohwinkel, Nana Burns, Ethan Coit, Xiaoyi Yuan, Eszter K. Vladar, Christina Sul, Eric P. Schmidt, Peter Carmeliet, Kurt Stenmark, Eva S. Nozik, Rubin M. Tuder, Holger K. Eltzschig

**Affiliations:** 1 Cardio Vascular Pulmonary Research Lab and; 2 Section of Critical Care Medicine, Department of Pediatrics, School of Medicine, University of Colorado, Aurora, Colorado, USA.; 3 Department of Anesthesiology, Critical Care and Pain Medicine, University of Texas Health Science Center Houston, Houston, Texas, USA.; 4 Program in Translational Lung Research, Division of Pulmonary Sciences and Critical Care Medicine, School of Medicine, University of Colorado, Aurora, Colorado, USA.; 5 Laboratory of Angiogenesis and Vascular Metabolism, Department of Oncology and Leuven Cancer Institute (LKI), KU Leuven, VIB Center for Cancer Biology, VIB, Leuven, Belgium.; 6 Laboratory of Angiogenesis and Vascular Heterogeneity, Department of Biomedicine, Aarhus University, Aarhus, Denmark.; 7 Center for Biotechnology, Khalifa University of Science and Technology, Abu Dhabi, United Arab Emirates.

**Keywords:** Metabolism, Pulmonology, Glucose metabolism, Hypoxia, Molecular biology

## Abstract

Acute lung injury (ALI) is a severe form of lung inflammation causing acute respiratory distress syndrome in patients. ALI pathogenesis is closely linked to uncontrolled alveolar inflammation. We hypothesize that specific enzymes of the glycolytic pathway could function as key regulators of alveolar inflammation. Therefore, we screened isolated alveolar epithelia from mice exposed to ALI induced by injurious ventilation to assess their metabolic responses. These studies pointed us toward a selective role for isoform 3 of the 6-phosphofructo-2-kinase/fructose-2,6-bisphosphatase (PFKFB3). Pharmacologic inhibition or genetic deletion of *Pfkfb3* in alveolar epithelia (*Pfkfb3^loxP/loxP^* SPC-ER-Cre^+^ mice) was associated with profound increases in ALI during injurious mechanical ventilation or acid instillation. Studies in genetic models linked Pfkfb3 expression and function to Hif1a. Not only did intratracheal pyruvate instillation reconstitute *Pfkfb3^loxP/loxP^* or *Hif1a^loxP/loxP^* SPC-ER-Cre^+^ mice, but pyruvate was also effective in ALI treatment of wild-type mice. Finally, proof-of-principle studies in human lung biopsies demonstrated increased PFKFB3 staining in injured lungs and colocalized PFKFB3 to alveolar epithelia. These studies reveal a specific role for PFKFB3 in counterbalancing alveolar inflammation and lay the groundwork for novel metabolic therapeutic approaches during ALI.

## Introduction

Acute lung injury (ALI) is a severe form of lung inflammation with its clinical manifestation as acute respiratory distress syndrome (ARDS) in patients (1, 2). ARDS is characterized by an acute onset of hypoxemia in conjunction with pulmonary edema, which is not explained entirely by fluid overload or cardiac disease (3). The ongoing COVID-19 pandemic highlights the impact of ARDS (2, 4). Moreover, studies demonstrated that ARDS survivors have significant long-term sequelae, which leads to increased usage of health care resources (5). Despite an improved understanding of ARDS pathogenesis, current therapies are mainly symptomatic, which accounts for the plateauing of mortality since the mid-1990s despite considerable advances in the study of ARDS (6, 7). Therefore, targeting novel therapeutic approaches to dampen or resolve (8, 9) alveolar inflammation as treatment approaches for ARDS are currently areas of intense research (10–13).

The alveolar epithelium is composed of type I and type II alveolar epithelial cells (14). Although alveolar type I cells comprise a higher percentage of the lung surface area, both cell types are found in similar numbers in the lung (15). Alveolar type II (ATII) cells play a critical part in ALI, as established in experimental models of lung insults (16). Recent studies have emphasized their critical role in repair processes of the disrupted epithelial surface, which are initiated immediately after epithelial injury (17). The alveolar epithelium has a critical role in coordinating alveolar inflammation responses (10, 11). Alveolar inflammation plays a key role in the pathogenesis of ALI (18). During alveolar inflammation, profound shifts in the supply and demand ratios for metabolites are known to occur (19, 20). Under normal circumstances, energy demands of pulmonary tissue compartments are met predominantly by oxidative phosphorylation. Alveolar epithelium is known for its capacity to divert pyruvate away from oxidative phosphorylation (12, 20, 21). This metabolic switch to high glycolytic rates and glucose dependency was first described by Otto Warburg and has gained significant attention in the context of cancer metabolism and hypoxia signaling (22). Emerging evidence indicates that the high demand for biosynthetic precursors as well as the increased energy demand of cells responding to infection or inflammation is in fact also met by a similar metabolic switch, for example in ARDS (21, 23, 24).

Several recent studies show that during inflammatory conditions such as occur during ARDS, alveolar epithelia can stabilize HIFs to optimize their carbohydrate metabolism (12) or to promote their ability to repair the injured alveolus following injury (25).

We proposed highly specific components of the glycolytic pathway could play selective and functionally critical roles during alveolar inflammation. Consistent with our hypothesis, we found that HIF1A drove the induction of isoform 3 of the 6-phosphofructo-2-kinase/fructose-2,6-bisphosphatase (PFKFB3), leading to an enhanced capacity for glycolysis as an endogenous protective pathway by which excessive alveolar inflammation is counterbalanced during ARDS.

## Results

### Alveolar epithelial levels of glycolytic enzymes and glycolysis intermediates are elevated during ALI.

**Figure 1:**
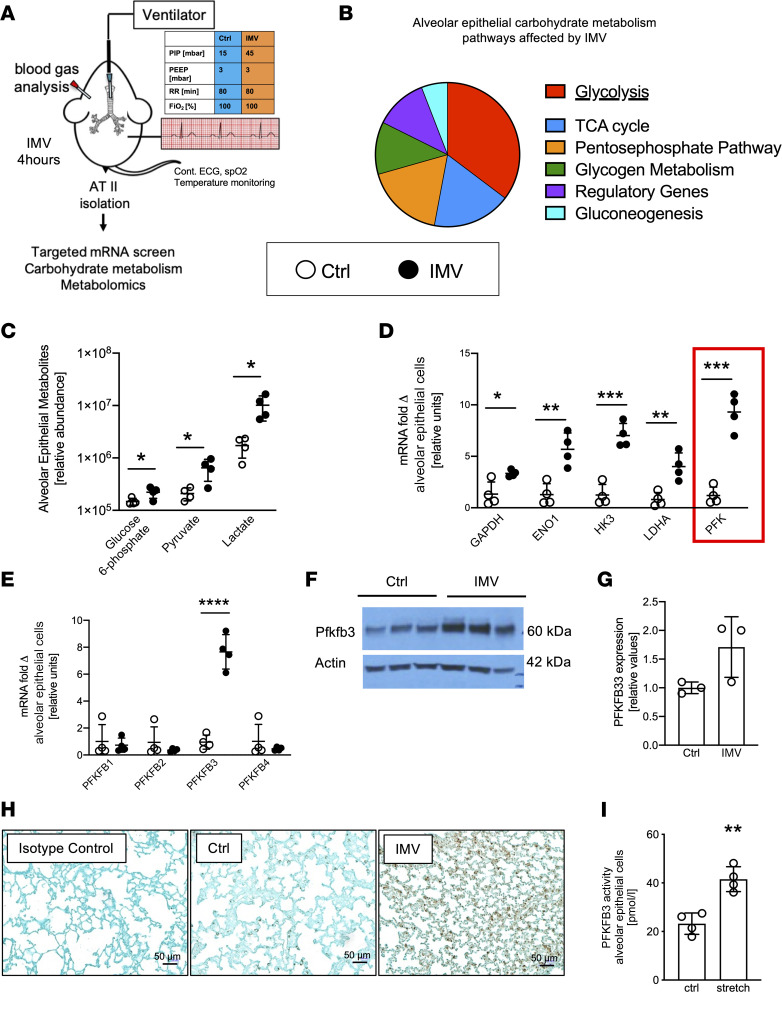
Glycolysis is upregulated in ATII cells in response to ALI. (**A**) Schematic overview of experiment: mice (matched for age, sex, and weight) were ventilated with the following parameters: peak inspiratory pressure (PIP) 45 mbar, respiratory rate (RR) 80, PEEP 3 mbar, and FiO_2_ 100% in injurious ventilation (IMV). Control group (Ctrl) animals were ventilated with the same parameters but with a PIP of 15 mbar. After ventilation for 4 hours alveolar epithelial cells were isolated for RNA or metabolomics analysis. spO_2_, oxygen saturation. (**B**) mRNA isolated from alveolar epithelial cells after IMV (*n* = 4) and Ctrl (*n* = 3) was analyzed using a targeted carbohydrate metabolism screen (80 genes). A total of 17 genes were found to be differentially regulated in the IMV group, and different pathways of carbohydrate metabolism were analyzed. Proportions of the pie diagram represent number of genes out of total significant genes. (**C**) Alveolar epithelial cells were isolated after animals were ventilated (*n* = 4/group), and glycolytic intermediates were determined by mass spectrometry. (**D**) Validation of the most differentially regulated genes in alveolar epithelial cells by qPCR (*n* = 4/group). (**E**) mRNA expression of PFKFB subunits was determined by qPCR (*n* = 4/group). (**F** and **G**) PFKFB3 protein levels in alveolar epithelial cells by Western blot (*n* = 3/group). (**H**) Immunohistochemical expression of PFKFB3 in mouse lungs after IMV and control ventilation. Rabbit IgG was used as a negative isotype control. (**I**) Measurement of PFKFB3 activity in alveolar epithelial MLE-12 cell line in response to cyclic stretch (*n* = 4/group). (**A**, **D**, and **E**) Six males and 5 females. (**C**) Four males and 4 females. (**F** and **G**) Two males and 4 females. (**H**) Two males and 2 females. GAPDH, glyceraldehyde-3-phosphate dehydrogenase; ENO1, enolase 1; HK3, hexokinase 3; LDHA, lactate dehydrogenase A; PFK, phosphofructokinase. Data are represented as mean ± SD. *P < 0.05, **P < 0.01, ***P < 0.001, ****P < 0.0001. Data were analyzed with 2-tailed, unpaired Student’s *t* test. Full, uncut gels are available in supplemental materials.

Previous studies suggested that alveolar epithelia undergo metabolic adaptation during stress conditions (12). Therefore, we performed studies to gain insight into specific checkpoints of alveolar epithelial carbohydrate metabolism during lung injury. Based on a previously described protocol (12, 26), we exposed mice for 4 hours to injurious mechanical ventilation (pressure-controlled ventilation, inspiratory pressures of 45 mbar at 100% fraction of inspired oxygen [FiO_2_]; positive end-expiratory pressure [PEEP] of 3 mbar) to induce ALI. We isolated ATII cells for molecular analysis of metabolic responses (Figure 1A). For controls, we used isolated alveolar epithelia from mice that were mechanically ventilated in a similar setting using pressure-controlled ventilation at 15 mbar. We performed a targeted quantitative PCR (qPCR) screen of carbohydrate metabolism (84 genes encoding key enzymes of carbohydrate metabolism). Genes encoding the glycolytic pathway experienced the highest degree of transcriptional induction of the metabolic pathways (Figure 1B). Measurements of metabolic intermediates using mass spectrometry supported very robust increases of glycolytic intermediates in murine primary alveolar epithelia exposed to ALI (Figure 1C). In addition, we exposed cultured murine primary alveolar epithelial cells to cyclic mechanical stretch — an in vitro model of alveolar injury resembling IMV (12). These in vitro studies of alveolar stretch injury confirmed robust increases in glycolytic intermediates, including glucose-6-phosphate, pyruvate, and lactate (Supplemental Figure 1A; supplemental material available online with this article; https://doi.org/10.1172/jci.insight.157855DS1). Taken together, these findings demonstrate that during ALI, alveolar epithelia increase transcription of their glycolytic enzymes and experience elevated concentrations of glycolysis intermediates in response to injury or stretch.

### Selective induction of the PFKFB3 iso-enzyme during ALI exposure.

We examined the relative contribution of the individual control points of the glycolytic pathway. We studied transcriptional changes of individual glycolytic enzymes in ATII cells of mice exposed to ALI. In agreement with our array studies (Figure 1B), we observed that the glycolytic enzymes glyceraldehyde-3-phosphate dehydrogenase (GAPDH), enolase (ENO), hexokinase 3 (HK3), and lactate dehydrogenase A (LDHA) were robustly induced. We found the most profound increase in transcript levels for phosphofructokinase (PFK) (Figure 1D). PFK catalyzes the phosphorylation of fructose-6-phosphate to fructose-1,6-bisphosphate and has been shown to function as a key regulator of the glycolytic pathway (27). PFK forms a regulatory subunit with phosphofructokinase-2/fructose-2,6-bisphosphatase (PFKFB) (28). PFKFB is known for its critical role in enhancing glycolytic flux rates by producing fructose-2,6-bisphosphate, an allosteric activator of glycolysis, leading to an increased activity of downstream glycolytic enzymes (29). Interestingly, we found that injurious mechanical ventilation was associated with a highly selective induction of PFKFB3 in ATII cells (Figure 1E). In contrast, expression of other PFKFB isoforms’ (PFKFB1, 2, or 4) transcription was unaltered in ATII cells isolated from mice exposed to ALI induced by IMV. In addition to transcriptional induction, PFKFB3 protein level (Figure 1, F–H) was increased after ALI induced by mechanical ventilation. Moreover, in vitro studies of cyclic mechanical stretch of murine alveolar epithelia (MLE-12) showed association with increased PFKFB3 activity (Figure 1I). We previously showed that succinate was protective in ALI (20), so we examined whether succinate supplementation could directly trigger PFKFB3 activation. However, we found that supplementation with cell-permeable dimethyl succinate did not affect PFKFB3 activity (Supplemental Figure 1B). Expanding our studies to an additional model of lung injury, we used acid-induced ALI, which recapitulates ALI secondary to gastric aspiration in human patients. For these studies, we instilled 50 μL of 0.125 M hydrochloric acid (HCl) intratracheally into the lungs of anesthetized mice and harvested the lungs after 3 days. Similar to findings in ALI induced by mechanical ventilation, we found selective induction of PFKFB3 transcript levels in ATII cells isolated 3 days after acid-induced ALI (Supplemental Figure 1C). Moreover, PFKFB3 protein levels were elevated 3 days after acid instillation by Western blot and immunohistological staining (Supplemental Figure 1, D and E). Taken together, these findings demonstrate a selective induction of alveolar epithelial PFKFB3 following cyclic mechanical stretch exposure in vitro, or during murine ALI induced by IMV or acid instillation in vivo.

### Pharmacologic inhibition of PFKFB3 is associated with increased alveolar inflammation.

**Figure 2:**
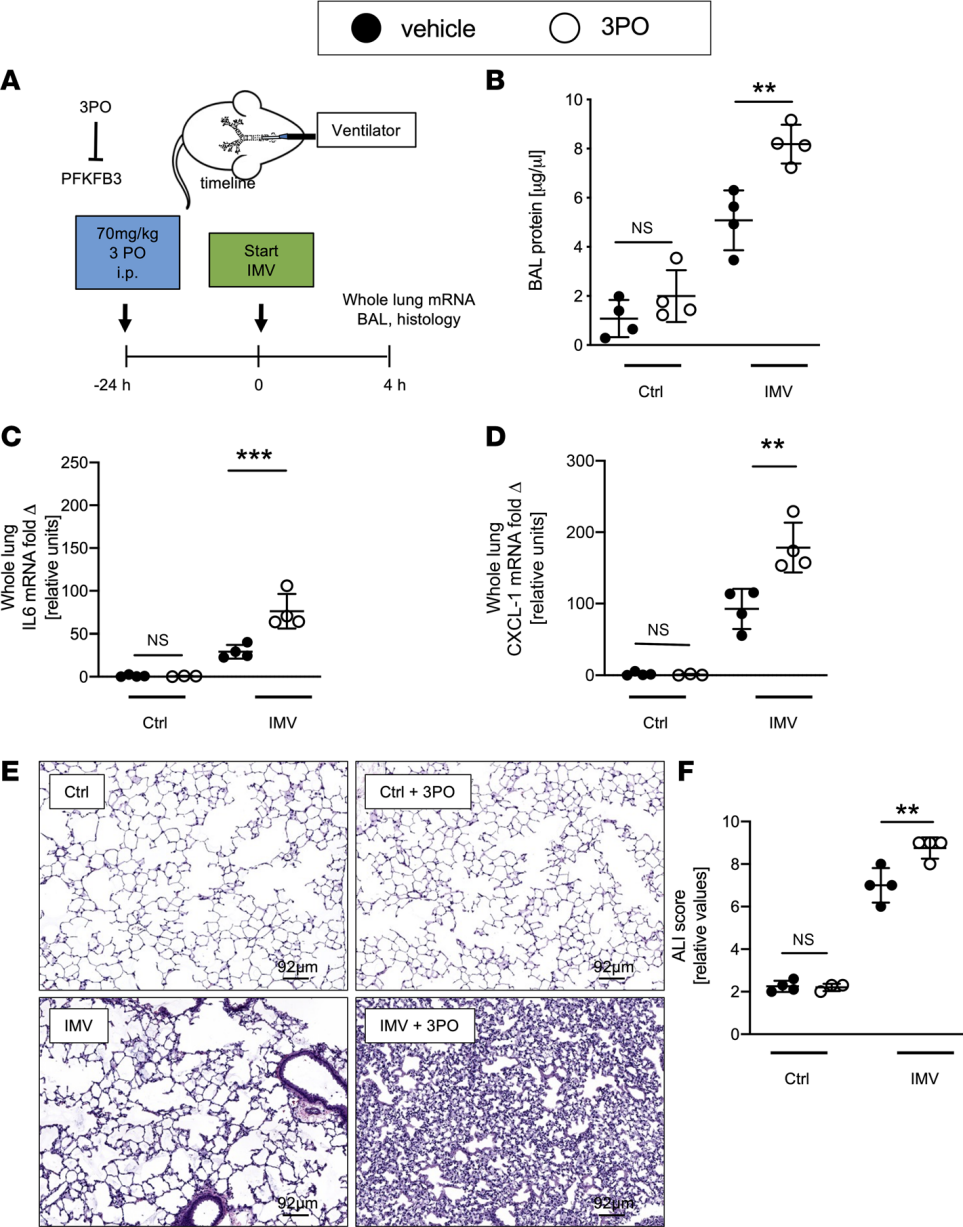
Pharmacological inhibition of PFKFB3 exacerbates lung injury. (**A**) Schematic of the experiment: C57BL/6 mice (matched for age, sex, and weight) received 70 mg/kg of PFKFB3 inhibitor 3PO i.p. 24 hours prior to ventilation, with the Ctrl group being ventilated with a PIP of 15 mbar and IMV group with a PIP of 45 mbar. The groups that were not treated with 3PO received vehicle (DMSO). After 4 hours of ventilation the lungs were removed. (**B**) BALF protein concentration was measured with Bradford Assay qPCR (*n* = 4/group). (**C** and **D**) IL-6 and CXCL1 mRNA expression in whole-lung tissue was determined by qPCR (*n* = 4/group). (**E** and **F**) Representative images of H&E-stained lungs and cumulative lung injury score, which is a combined score of cellular infiltrates, interstitial congestion and hyaline membrane formation, and hemorrhage (*n* = 4 each IMV, IMV+3PO, Ctrl groups; *n* = 3 Ctrl+3PO group). (**B**) Eight males and 8 females. (**C**–**F**) Seven males and 8 females. Data are represented as mean ± SD. **P < 0.01, ***P < 0.001. Data were analyzed with 1-way ANOVA with Tukey’s correction for multiple comparisons.

We investigated whether pharmacologic PFKFB3 inhibition affects lung inflammation and outcomes in ALI models. We treated wild-type mice with the PFKFB3 inhibitor 3-(3-pyridinyl)-1-(4-pyridinyl)-2-propen-1-one (3PO) (30). Based on previous studies (30), mice received 70 mg/kg 3PO i.p. 24 hours prior to initiation of ALI by IMV (Figure 2A). We found that mice treated with 3PO experienced exacerbation of lung injury, including increased protein levels in the bronchoalveolar lavage fluid (BALF) and elevated mRNA expression of proinflammatory cytokines IL-6 and CXCL1 in their lungs (Figure 2, B–D), leading to exacerbated histologic lung injury and histologic ALI scores (Figure 2, E and F). Similarly, we observed that mice subjected to acid aspiration–induced ALI experienced a more severe form of ALI when pretreated with 3PO 24 hours prior to induction of ALI as compared with vehicle control treatment (Supplemental Figure 2, A–F). Taken together, these pharmacologic studies support that PFKFB3 activation protects against ALI.

### Pfkfb3^loxP/loxP^ SPC-Cre-ER^+^ mice experience more severe ALI during IMV or following acid aspiration.

**Figure 3:**
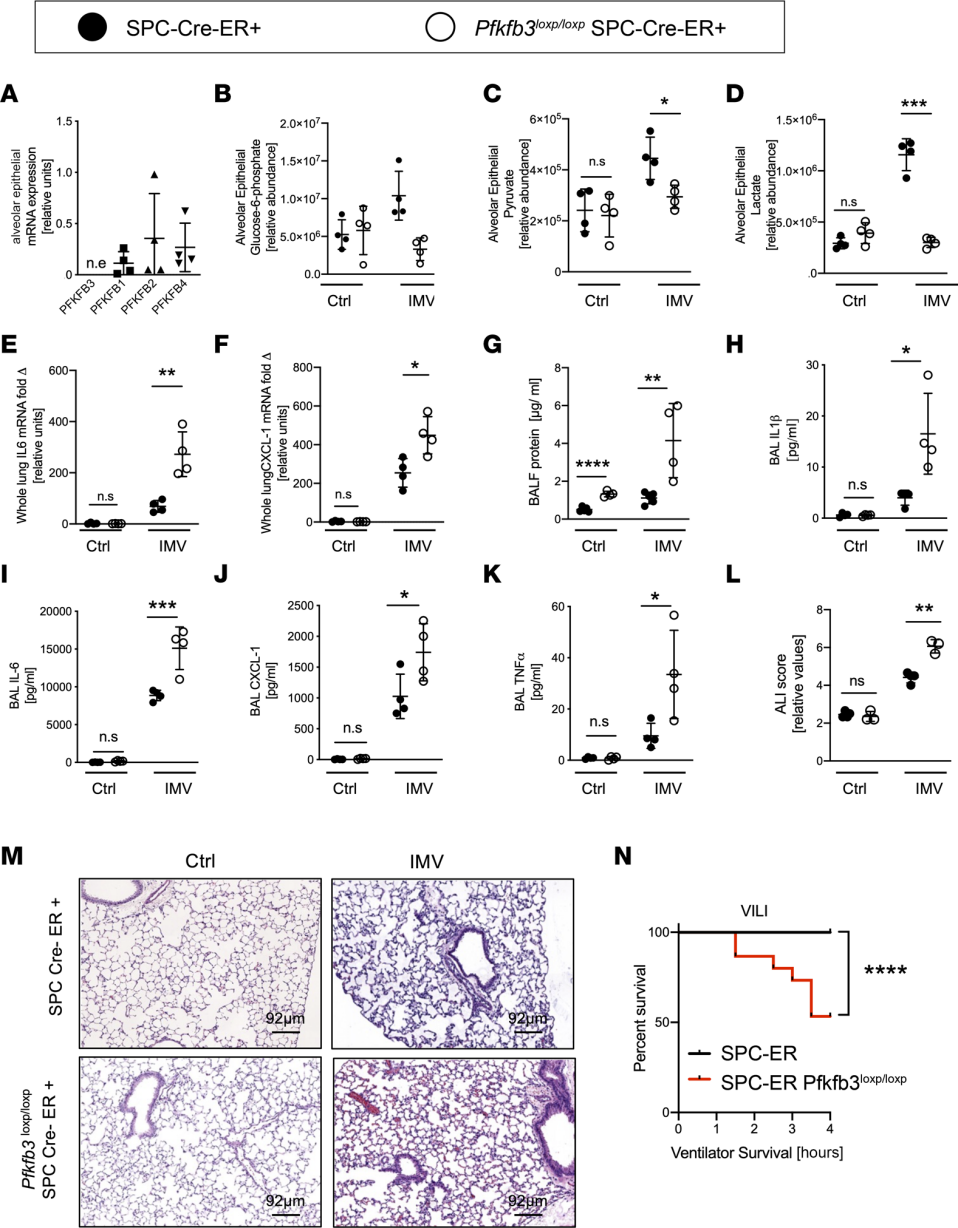
Functional consequences of ATII specific deletion of *Pfkfb3* in lung injury induced by injurious ventilation. *Pfkfb3^loxP/loxP^* Surfactant Cre^+^ (*Pfkfb3^loxP/loxP^* SPC-ER-Cre^+^) mice and controls (SPC-ER-Cre^+^) were exposed to injurious (IMV, PIP 45 mbar) or control ventilation (Ctrl, PIP 15 mbar) (age, sex, and weight matched). (**A**) Alveolar epithelial cells were isolated from *Pfkfb3^loxP/loxP^* SPC-ER-Cre^+^ mice, and PFKFB isoform mRNA expression was determined via qPCR (*n* = 4/group). (**B**–**D**) Glycolytic intermediates from alveolar epithelial cells isolated from *Pfkfb3^loxP/loxP^* SPC-ER-Cre^+^ and SPC-ER-Cre^+^ after IMV and control ventilation were determined with mass spectrometry (*n* = 4/group). (**E** and **F**) IL-6 and CXCL1 mRNA expression in whole-lung tissue was determined by qPCR (*n* = 4/group). (**G**) Protein concentration was measured in BALF with Bradford Assay (*n* = 4 SPC-ER-Cre^+^, *n* = 5 *Pfkfb3^loxP/loxP^* SPC-ER-Cre^+^ group). (**H**–**K**) Concentration of cytokines in BALF was measured by ELISA (*n* = 4/group). (**L** and **M**) Representative images of H&E-stained lungs and cumulative lung injury score, which is a combined score of cellular infiltrates, interstitial congestion and hyaline membrane formation, and hemorrhage (*n* = 3 SPC-ER-Cre^+^, *n* = 4 *Pfkfb3^loxP/loxP^* SPC-ER-Cre^+^ group). (**N**) Survival curve in response to IMV for *Pfkfb3^loxP/loxP^* SPC-ER-Cre^+^ and SPC-ER-Cre^+^. (**A**) Four males and 4 females. (**B**–**D**) Eight males and 8 females. (**F** and **G**) Eight males and 8 females. (**E** and **H**–**K**) Eight males and 8 females. (**L** and **M**) Eight males and 6 females. (**N**) Thirty-one males and 31 females. Survival curve was analyzed with log-rank (Mantel-Cox) test. Other data are represented as mean ± SD and were analyzed with 1-way ANOVA with Tukey’s correction for multiple comparisons, *P < 0.05, **P <0.01, ***P < 0.001, ****P < 0.0001. n.e., not expressed.

We next pursued studies using a genetic model that would allow us to determine the functional contribution of ATII cell–specific PFKFB3 during ALI. Since germline deletion of PFKFB3 is associated with embryonic lethality (31), we applied a selective deletion of *Pfkfb3* specifically in ATII cells in adult mice. We crossbred previously described Pfkfb^3tm1Pec^ (*Pfkfb3^loxP/loxP^*) mice (32) with an ATII cell–specific driver line [Sftpc^tm1(cre/ERT2)Blh^; SPC-Cre-ER^+^] (Supplemental Figure 3A) (32). Successful deletion of PFKFB3 protein was confirmed in isolated alveolar epithelia of *Pfkfb3^loxP/loxP^* SPC-Cre-ER^+^ mice following tamoxifen treatment when compared with SPC-Cre-ER^+^ control mice by Western blotting (Supplemental Figure 3B). When housed in a pathogen-free animal facility, *Pfkfb3^loxP/loxP^* SPC-Cre-ER^+^ mice were of normal weight (Supplemental Figure 3C), had normal-sized litters, and did not display any apparent immunologic deficits or malformations. Analysis of relative expression levels of *Pfkfb* isoforms in isolated ATII cells demonstrated the successful deletion of *Pfkfb3*, while other *Pfkfb* isoforms (*Pfkfb1*, *Pfkfb2*, or *Pfkfb4*) were unaltered in their expressional profile (Figure 3A).

We exposed *Pfkfb3^loxP/loxP^* SPC-Cre-ER^+^ mice or littermate SPC-Cre-ER^+^ controls matched in age, sex, and weight to ALI induced by IMV following tamoxifen induction of Cre-recombinase. First, the analysis of glycolytic metabolites in isolated ATII cells revealed no differences between alveolar epithelia isolated from *Pfkfb3^loxP/loxP^* SPC-Cre-ER^+^ mice or controls at baseline (Figure 3, B–D). However, the increased levels of the glycolysis intermediates glucose-6-phosphate, pyruvate, or lactate levels in response to injury in control mice were completely abolished in alveolar epithelia isolated from *Pfkfb3^loxP/loxP^* SPC-Cre-ER^+^ mice (Figure 3, B–D). Moreover, we observed that *Pfkfb3^loxP/loxP^* SPC-Cre-ER^+^ mice exhibited a far more severe phenotype during ALI as compared with control mice, as evidenced by a greater increase in BALF protein content (Figure 3E), higher elevations of the transcript levels of IL-6 and CXCL1 in their lungs (Figure 3, F and G), and higher levels of proinflammatory cytokines in the BALF (Figure 3, H–K). We did not find an apparent difference in lung inflammation comparing male or female *Pfkfb3^loxP/loxP^* SPC-Cre-ER^+^ mice (Supplemental Figure 3, D and E), so it appears that sex as a biological variable does not play a major role in this response. Furthermore, the histologic analysis of ALI-associated alveolar injury revealed that *Pfkfb3^loxP/loxP^* SPC-Cre-ER^+^ mice experienced a more severe form of histologic ALI and ALI scoring compared with controls (Figure 3, L and M). Of note, in response to ALI induced by mechanical ventilation, *Pfkfb3^loxP/loxP^* SPC-Cre-ER^+^ mice frequently showed worse alveolar hemorrhage than the SPC-Cre-ER^+^ controls. Finally, *Pfkfb3^loxP/loxP^* SPC-Cre-ER^+^ mice when subjected to ALI induced by mechanical ventilation displayed a marked decrease in survival time compared with control SPC-Cre-ER^+^ mice (Figure 3N). Together these findings provide what may be the first genetic evidence for a protective role for *Pfkfb3* expressed in alveolar epithelia in murine models of ARDS.

**Figure 4:**
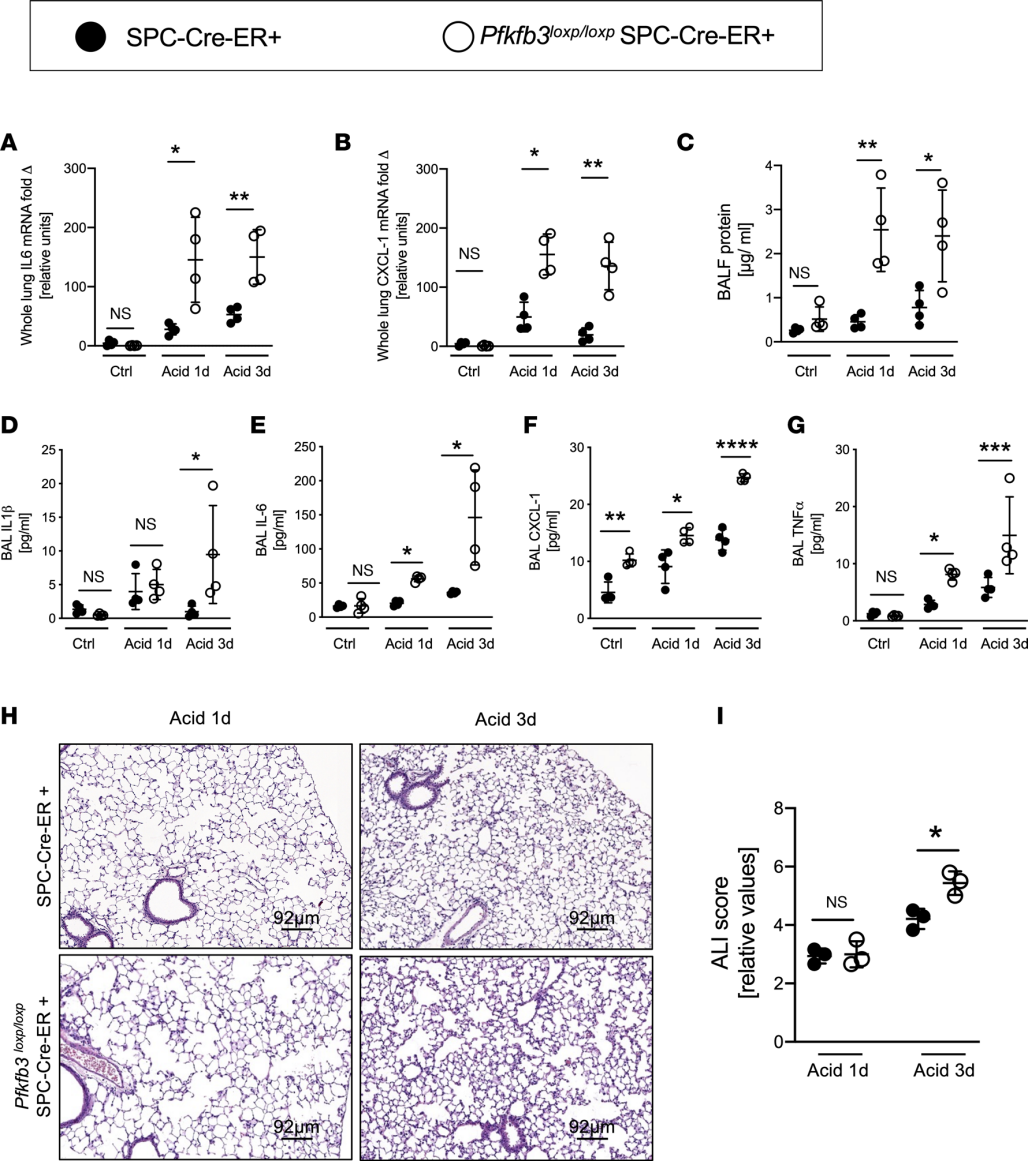
Functional consequences of ATII specific deletion of *Pfkfb3* in acid-induced ALI. Lung injury was induced in *Pfkfb3^loxP/loxP^* SPC-ER-Cre^+^ mice and age-, sex-, and weight-matched controls (SPC-ER-Cre^+^) by acid instillation with i.t. HCl. The control groups received pH-controlled NaCl. After 1 and 3 days, the lungs were removed. (**A** and **B**) IL-6 and CXCL1 mRNA expression in whole-lung tissue was determined by qPCR (*n* = 4/group). (**C**) Protein concentration measured in BALF with Bradford Assay (*n* = 4/group). (**D**–**G**) Concentration of cytokines in BALF was determined by ELISA (*n* = 4/group). (**H** and **I**) Representative images of H&E-stained lungs and cumulative lung injury score, which is a combined score of cellular infiltrates, interstitial congestion and hyaline membrane formation, and hemorrhage (*n* = 3/group). (**A**–**G**) Eleven males and 13 females. (**H** and **I**) Six males and 6 females. Data are represented as mean ± SD. *P < 0.05, **P < 0.01, ***P < 0.001, ****P < 0.0001. Data were analyzed with 1-way ANOVA with Tukey’s correction for multiple comparisons.

We exposed *Pfkfb3^loxP/loxP^* SPC-Cre-ER^+^ mice or corresponding littermate SPC-Cre-ER^+^ controls matched by age, sex, and weight to acid-induced ALI, following tamoxifen-mediated induction of Cre-recombinase. Similar to the above studies in ALI induced by mechanical ventilation, we found that *Pfkfb3^loxP/loxP^* SPC-Cre-ER^+^ mice showed a far more severe phenotype of alveolar inflammation and lung injury as compared with controls. *Pfkfb3^loxP/loxP^* SPC-Cre-ER^+^ mice experienced increased albumin leakage into their BALF after 1 or 3 days following i.t. instillation of HCl. Similarly, production of proinflammatory cytokines’ transcript levels (Figure 4, A–C) was increased in the lungs. Also, proinflammatory cytokine release into the BALF (Figure 4, D–G) was elevated. Moreover, we observed a more severe degree of alveolar inflammation and lung injury as evidenced on representative histologic slides (Figure 4H), verified by blinded scoring for lung injury severity in *Pfkfb3^loxP/loxP^* SPC-Cre-ER^+^ mice (Figure 4I). Additionally, we investigated whether alveolar epithelial deletion of *Pfkfb3* affects the recruitment of inflammatory cells to the lung. We found no difference in neutrophil and monocyte/macrophage recruitment to the BALF in both IMV-induced and acid-induced lung injury in *Pfkfb3^loxP/loxP^* SPC-ER-Cre^+^ mice compared to SPC-ER-Cre^+^ controls (Supplemental Figure 3, F and G).

Taken together, these studies provide genetic in vivo evidence for a critical role of alveolar specific *Pfkfb3* in dampening alveolar inflammation during ALI induced by detrimental mechanical ventilation or during acid aspiration.

### Identification of alveolar epithelial Hif1a as an upstream regulator of Pfkfb3-mediated lung protection.

**Figure 5:**
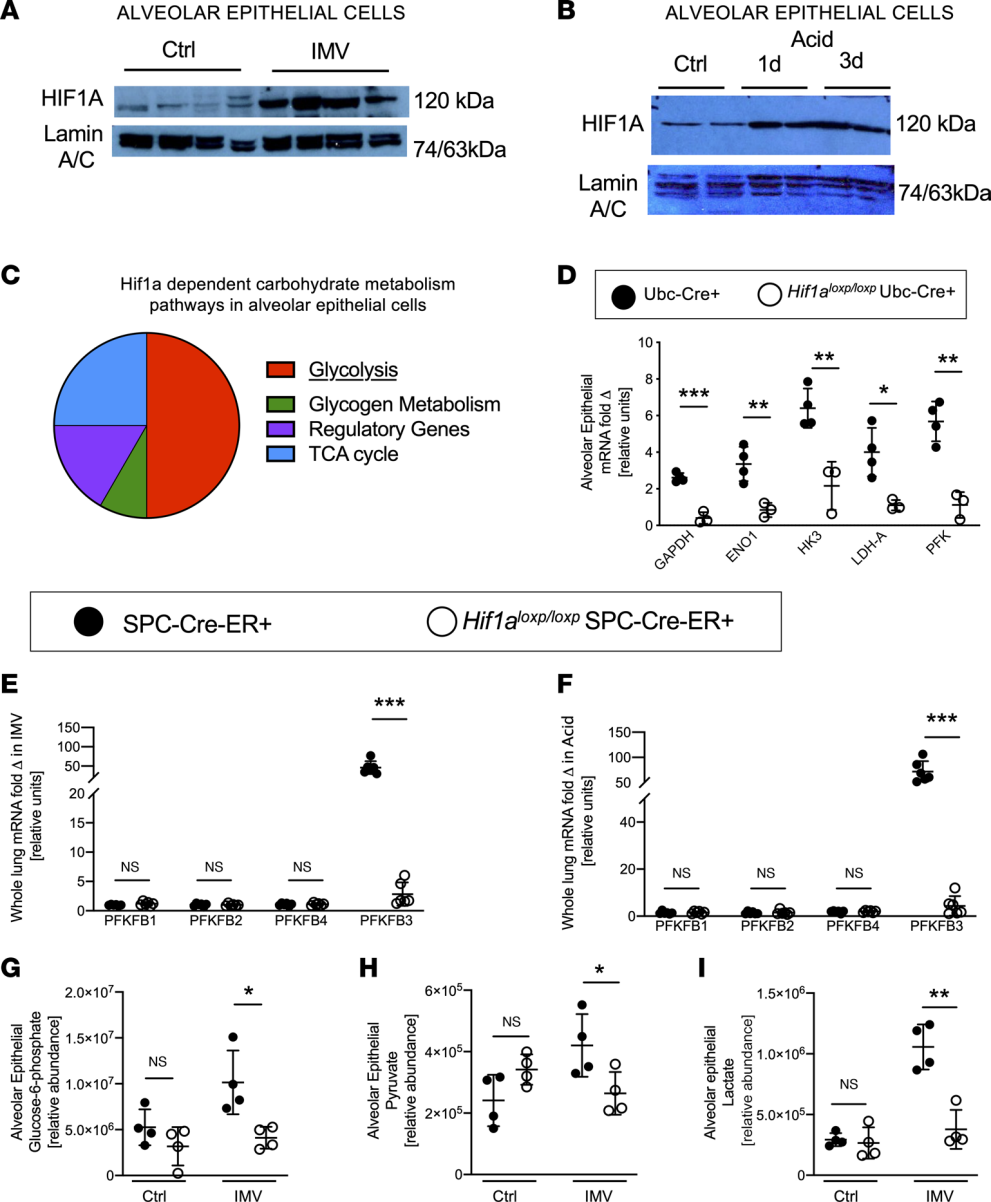
Alveolar epithelial glycolysis induced by ALI is controlled by HIF1A. (**A** and **B**) ALI was induced in C57BL/6 mice (matched for age, sex, and weight) by IMV (**A**) or acid instillation (**B**). Alveolar epithelial cells were isolated and HIF1A protein expression was determined in nuclear fraction via Western blot (each blot lane is from alveolar epithelial cells of an individual animal). (**C**) mRNA was isolated from alveolar epithelial cells isolated from *Hif1a^loxP/loxP^* Ubc-Cre^+^ mice (*n* = 3/group) and Ubc-Cre^+^ (*n* = 4/group) control mice after IMV, and a targeted carbohydrate metabolism screen (80 genes) was performed. A total of 12 genes were found to be differentially regulated in the Ubc-Cre^+^ mice, and different pathways of carbohydrate metabolism were analyzed. Proportions of the pie diagram represent number of genes out of total significant genes. (**D**) Validation of the most differentially regulated genes in alveolar epithelial cells and LDHA by qPCR (*n* = 3 *Hif1a^loxP/loxP^* Ubc-Cre^+^ group and *n* = 4 Ubc-Cre^+^ group). (**E** and **F**) Pfkfb3 mRNA expression was measured in whole lungs from *Hif1a^loxP/loxP^* SPC-ER-Cre^+^ and SPC-ER-Cre^+^ mice after IMV and acid instillation (*n* = 6/group). (**G**–**I**) Isolation of alveolar epithelial cells from *Hif1a^loxP/loxP^* SPC-ER-Cre^+^ and SPC-ER-Cre^+^ after IMV and control ventilation followed by determination of glycolytic intermediates with mass spectrometry (*n* = 4/group, except *Hif1a^loxP/loxP^* SPC-ER-Cre^+^ group *n* = 3). (**A** and **B**) Seven males and 7 females. (**C** and **D**) Nine males and 11 females. (**E** and **F**) Seventeen males and 19 females. Data are represented as mean ± SD. *P < 0.05, **P < 0.01, ***P < 0.001. Data were analyzed with 2-tailed, unpaired Student’s *t* test. Full, uncut gels are available in supplemental materials.

Previous studies had shown that the *Pfkfb3* gene contains a promotor sequence for HIF1A (33) and established a functional role for HIF1A as a regulator of the glycolytic pathway, including PFKFB3, under conditions of limited oxygen availability (19, 34). To examine the potential functional role of HIF1A in regulating PFKFB3 during alveolar inflammation, we first performed studies to address alveolar epithelial HIF1A protein stabilization during ALI. For this purpose, we isolated alveolar epithelial cells after ALI induced by either mechanical ventilation or acid aspiration and examined them for HIF stabilization. Consistent with previous studies of alveolar epithelia exposed to cyclic mechanical stretch, or whole lungs of mice exposed to ALI (12), we observed robust stabilization of HIF1A protein in the nuclear fraction derived from alveolar epithelial cell lysate in response to ALI (Figure 5, A and B). We performed targeted mRNA screening of carbohydrate metabolism to examine a role of HIF1A in regulating specific glycolytic enzymes in alveolar epithelial cells during ALI. For this purpose, we used previously described mice with tamoxifen-inducible deletion of Hif1a (B6.129-Hif1a^tm3Rsjo/J^ UBC-cre/ERT2; *Hif1a^loxP/loxP^* Ubc-Cre^+^ mice) (35), since mice with homozygous deletion of *Hif1a* die during early embryogenesis (36). Ubc-Cre^+^ mice matched in age, sex, and weight were used as controls following tamoxifen-induced Cre stabilization (37). To address a potential role for alveolar epithelial Hif1a in regulating PFKFB3 during ALI, we subjected the mice to ALI induced by IMV over 4 hours and subsequently isolated alveolar epithelia. Pathway analysis demonstrated that the majority of differentially regulated genes were regulators of glycolysis (Figure 5C). Furthermore, a subsequent analysis using real-time PCR demonstrated that specific glycolytic enzymes identified in wild-type mice (Figure 1D) failed to upregulate in response to IMV in the *Hif1a^loxP/loxP^* Ubc-Cre^+^ mice (Figure 5D).

We next performed studies in mice with *Hif1a* deletion specifically in alveolar epithelial cells of the lungs (20). We had previously generated mice with alveolar specific deletion of *Hif1a* (*Hif1a^loxP/loxP^* SPC-Cre-ER^+^) and found that they were more susceptible to ALI induced by injurious ventilation (20). Consistent with those previously published findings, we showed here that when we exposed *Hif1a^loxP/loxP^* SPC-Cre-ER^+^ mice to ALI induced by instillation of HCl, the mice experienced more severe lung inflammation (Supplemental Figure 4, A and B) and more severe histologic tissue injury and ALI scoring (Supplemental Figure 4, C and D). We examined *Pfkfb3* transcript levels in *Hif1a^loxP/loxP^* SPC-Cre-ER^+^ mice in response to ALI induced by either mechanical ventilation or acid aspiration. These studies revealed that increases in *Pfkfb3* transcript levels with ALI induced by mechanical ventilation or acid instillation were completely abolished in alveolar epithelia isolated from *Hif1a^loxP/loxP^* SPC-Cre-ER^+^ mice compared with controls (Figure 5, E and F). Similarly, elevations of the glycolytic intermediates glucose-6-phosphate, pyruvate, and lactate were dampened in alveolar epithelia isolated from *Hif1a^loxP/loxP^* SPC-Cre-ER^+^ mice exposed to ALI (Figure 5, G–I). Taken together, these findings indicate that PFKFB3 is transcriptionally regulated by HIF1A during ALI, and functions to enhance the glycolytic capacity of alveolar epithelia, thereby dampening alveolar inflammation.

### Reconstitution of mice with alveolar epithelial deletion of Pfkfb3 or Hif1a.

**Figure 6:**
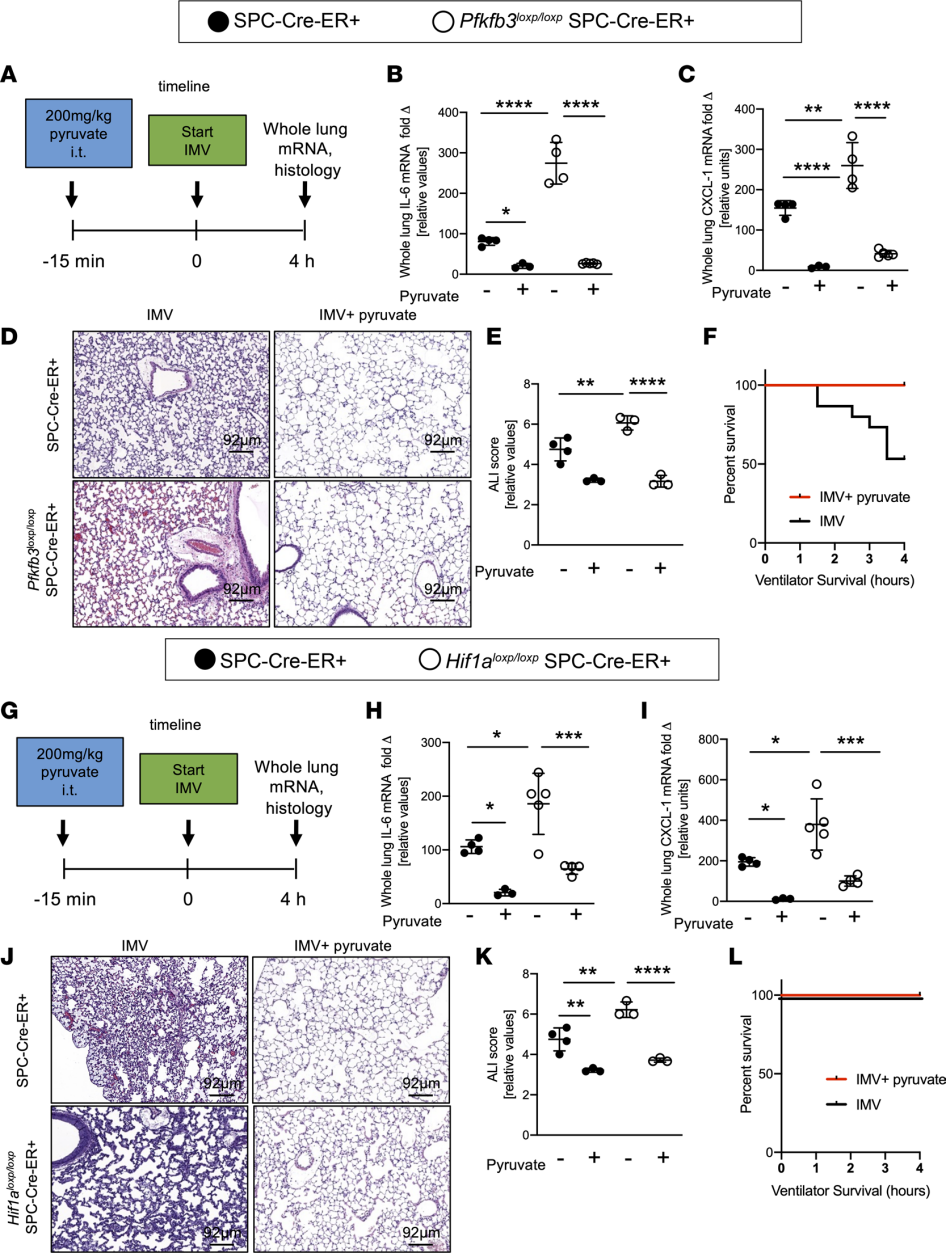
Locally delivered pyruvate reconstitutes *Hif1a^loxP/loxP^* SPC-ER-Cre^+^ and *Pfkfb3^loxP/loxP^* SPC-ER-Cre^+^ animals. (**A** and **G**) Schematic of experiment: C57BL/6 wild-type mice with matched weight and sex were used in all experiments. Alveolar epithelial cell–specific conditional knockout mice (*Hif1a^loxP/loxP^* SPC-ER-Cre^+^ and *Pfkfb3^loxP/loxP^* SPC-ER-Cre**^+^**) or control animals (SPC-ER-Cre**^+^**) received 200 mg/kg i.t. pyruvate 15 minutes prior to induction of IMV. After 4 hours lung tissue was harvested for analysis. (**B**, **C**, **H**, and **I**) IL-6 and CXCL1 mRNA expression was determined in whole-lung tissue by qPCR (*n* = 4/group, except SPC-ER-Cre^+^ pyruvate *n* = 3/group). (**D**, **E**, **J**, and **K**) Representative images of H&E-stained lungs from mice subjected to IMV and controls and cumulative lung injury score, which is a combined score of cellular infiltrates, interstitial congestion and hyaline membrane formation, and hemorrhage (*n* = 3/group, except SPC-ER-Cre^+^ IMV *n* = 4/group). The same SPC-ER-Cre^+^ control mice were used for histologic controls (**D**, **E**, **I**, and **J**). Survival curve in response to IMV with and without i.t. pyruvate treatment (**F** and **L**). (**B** and **C**) Eleven males and 15 females. (**D** and **E**) Thirteen males and 17 females. (**G** and **H**) Twelve males and 13 females. Data are represented as mean ± SD. *P < 0.05, **P < 0.01, ***P < 0.001, ****P < 0.0001. Data were analyzed with 1-way ANOVA with Tukey’s correction for multiple comparisons.

As proof of principle for the assertion that PFKFB3-driven glycolysis in alveolar epithelia plays a functional role in alveolar inflammation during ARDS, we first pursued studies to reconstitute the phenotype we had observed in *Pfkfb3^loxP/loxP^* SPC-Cre-ER^+^ mice. PFKFB3 is a critical downstream regulator of glycolysis, and *Pfkfb3^loxP/loxP^* SPC-Cre-ER^+^ mice showed decreased glycolytic intermediates downstream of PFKFB3 in alveolar epithelial cells (Figure 3, C and D). For these studies, we used the glycolysis intermediate pyruvate, which we had found to be deficient in *Pfkfb3^loxP/loxP^* SPC-Cre-ER^+^ mice exposed to ALI induced by mechanical ventilation (Figure 3C). Moreover, previous studies that used systemic treatment approaches with pyruvate in mice to induce torpor showed that pyruvate treatment was well tolerated by the experimental animals (38). In order to achieve delivery to alveolar epithelia, we chose the i.t. application route. We used a dose of 200 mg/kg of body weight i.t. (highest possible concentration that could be dissolved in 50 μL, which is the maximum i.t. instillation volume that is well tolerated; ref. 39) 15 minutes before inducing ALI by IMV in *Pfkfb3^loxP/loxP^* SPC-Cre-ER^+^ mice or littermate Cre^+^ controls matched in age, sex, and weight following tamoxifen induction (Figure 6A). Treatment-free control mice received an equal volume of vehicle, which was pH controlled to that of pyruvate. These studies demonstrated that pyruvate normalized the phenotype of *Pfkfb3^loxP/loxP^* SPC-Cre-ER^+^ mice, with attenuated lung inflammation (IL-6 and CXCL1, Figure 6, B and C) and attenuated histologic tissue injury and ALI scores (Figure 6, D and E). Moreover, these studies demonstrated therapeutic effects in control animals, demonstrating improvements in all of the above assessments of ALI, including improved survival time on the ventilator (Figure 6, B–F), suggesting that i.t. treatment with pyruvate attenuates alveolar inflammation during ALI.

We also performed reconstitution studies using i.t. pyruvate treatment of *Hif1a^loxP/loxP^* SPC-ER-Cre^+^ mice. Similar to mice with alveolar epithelial deletion of *Pfkfb3*, *Hif1a^loxP/loxP^* SPC-ER-Cre^+^ mice had more severe alveolar inflammation and ALI (Supplemental Figure 4) (20) and failed to elevate pyruvate levels in response to IMV (Figure 5H). Similar to the above studies in mice with alveolar deletion of *Pfkfb3*, *Hif1a^loxP/loxP^* SPC-ER-Cre^+^ mice or SPC-ER-Cre^+^ controls received 200 mg/kg body weight of i.t. pyruvate prior 15 minutes before the onset of ALI induction by IMV or vehicle (Figure 6G). These studies revealed a normalization of lung inflammation with decreased transcript levels for IL-6 and CXCL1 (Figure 6, H and I) and concomitantly improved ALI histologic score (Figure 6, J and K). In addition to the reconstitution of *Hif1a^loxP/loxP^* SPC-ER-Cre^+^ mice, we confirmed the treatment effects in control animals, thereby confirming the therapeutic effects of i.t. pyruvate during alveolar inflammation. Interestingly, unlike the *Pfkfb3^loxP/loxP^* SPC-ER-Cre^+^ mice, the *Hif1a^loxP/loxP^* SPC-ER-Cre^+^ mice did not display increased mortality in response to IMV (Figure 6L). Of note, in both *Hif1a^loxP/loxP^* SPC-ER-Cre^+^ and *Pfkfb3^loxP/loxP^* SPC-ER-Cre^+^ mice, inflammatory gene expression remained above the level of pyruvate-treated SPC-Cre-ER^+^ control mice. One potential reason for i.t. pyruvate only partially rescuing cytokine expression in the *Pfkfb3^loxP/loxP^* and *Hif1a^loxP/loxP^* SPC-ER-Cre^+^ mice could be that the *Pfkfb3^loxP/loxP^* and *Hif1a^loxP/loxP^* SPC-ER-Cre^+^ mice had exacerbated lung injury with an increased capillary leak, which leads to rapid diffusion of i.t. applied pyruvate in the lung interstitium and therefore decreased availability of pyruvate for the alveolar epithelium. This is in line with our finding that whole-lung homogenate from *Pfkfb3^loxP/loxP^* and *Hif1a^loxP/loxP^* SPC-ER-Cre^+^ mice had a higher pyruvate content compared with the SPC-ER-Cre^+^ controls. Conversely, BALF from SPC-ER-Cre^+^ controls had higher pyruvate levels than *Pfkfb3^loxP/loxP^* and *Hif1a^loxP/loxP^* SPC-ER-Cre^+^ mice (Supplemental Figure 5).

Taken together, these studies demonstrate that PFKFB3 and HIF1A resemble critical control points for alveolar inflammation during ARDS and indicate the likelihood that the observed phenotypes in mice deficient in alveolar epithelial *Pfkfb3* or *Hif1a* are related to their inability to increase glycolytic responses during ALI.

### Treatment with i.t. pyruvate after the onset of injury attenuates inflammation in ALI.

**Figure 7:**
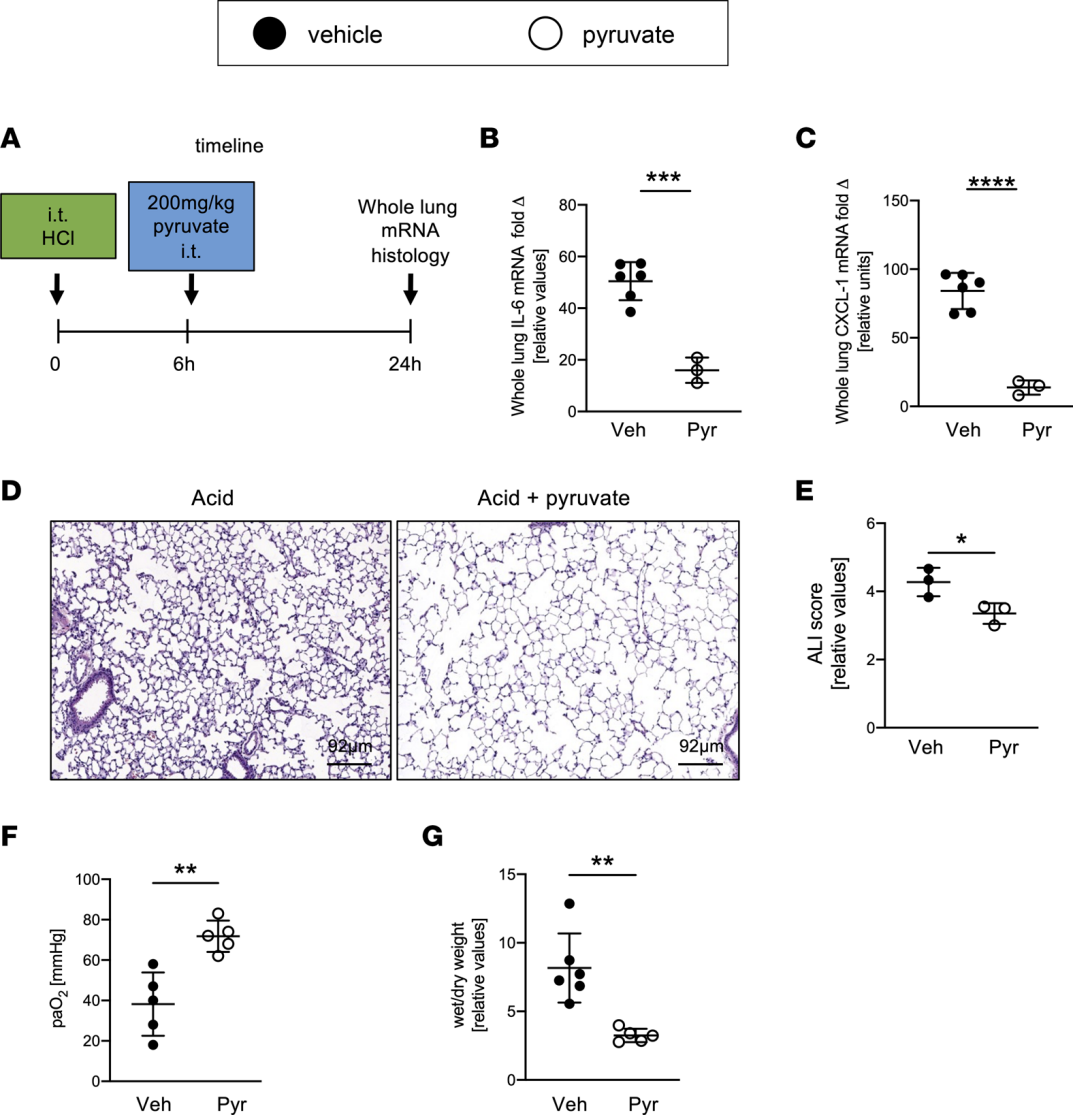
Locally delivered pyruvate is therapeutic in acid-induced lung injury. (**A**) Schematic of experiment. C57BL/6 mice were matched for age, weight, and sex. ALI was induced by acid instillation. The mice in the treatment group received 200 mg/kg i.t. pyruvate 6 hours after induction of lung injury (Pyr). Control animals received pH-controlled PBS (Ctrl). Lung tissue was harvested for analysis 24 hours after induction of ALI. (**B** and **C**) IL-6 and CXCL1 mRNA expression in whole lung tissue was determined with qPCR (*n* = 5 in control group, *n* = 3 in pyruvate group). (**D** and **E**) Representative images of H&E-stained lungs and cumulative lung injury score, which consists of a combined score of cellular infiltrates, interstitial congestion and hyaline membrane formation, and hemorrhage (*n* = 3/group). (**F**) Partial pressure of arterial oxygen (paO_2_) was measured from blood samples obtained from the aorta, *n* = 5/group. (**G**) Wet/dry ratio was calculated 4 days after instillation. (**B** and **C**) Five males and 4 females. (**D** and **E**) Three males and 3 females. (**E** and **F**) Five males and 6 females. Data are represented as mean ± SD. *P < 0.05, **P < 0.01, ***P < 0.001, ****P < 0.0001. Data were analyzed with 2-tailed, unpaired Student’s *t* test.

Having shown effective reconstitution of mice with alveolar epithelial *Pfkfb3* or *Hif1a* deletion with pyruvate treatment, we next extended those studies into a longer lasting ALI model, as the acid-induced ALI model enabled us to treat the mice after the onset of ALI. Six hours after acid instillation, we treated littermate mice matched in age, sex, and weight with i.t. pyruvate (200 mg/kg body weight) or an equal amount of vehicle control (pH controlled to pyruvate) in controls (Figure 7A). When analyzing the lungs 24 hours after acid aspiration for lung inflammation or ALI histology, we found attenuated transcript levels of IL-6 or CXCL1 (Figure 7, B and C) in conjunction with attenuated histologic lung injury or a blinded analysis of lung injury scores (Figure 7, D and E). Additionally, animals treated with i.t. pyruvate showed improved oxygenation and improved wet/dry ratio (Figure 7, F and G). Together, these studies indicate that treatment with i.t. pyruvate after the onset of acid-induced lung injury promotes attenuation of inflammation in ALI.

### PFKFB3 is elevated in alveolar epithelial cells during human ARDS.

**Figure 8:**
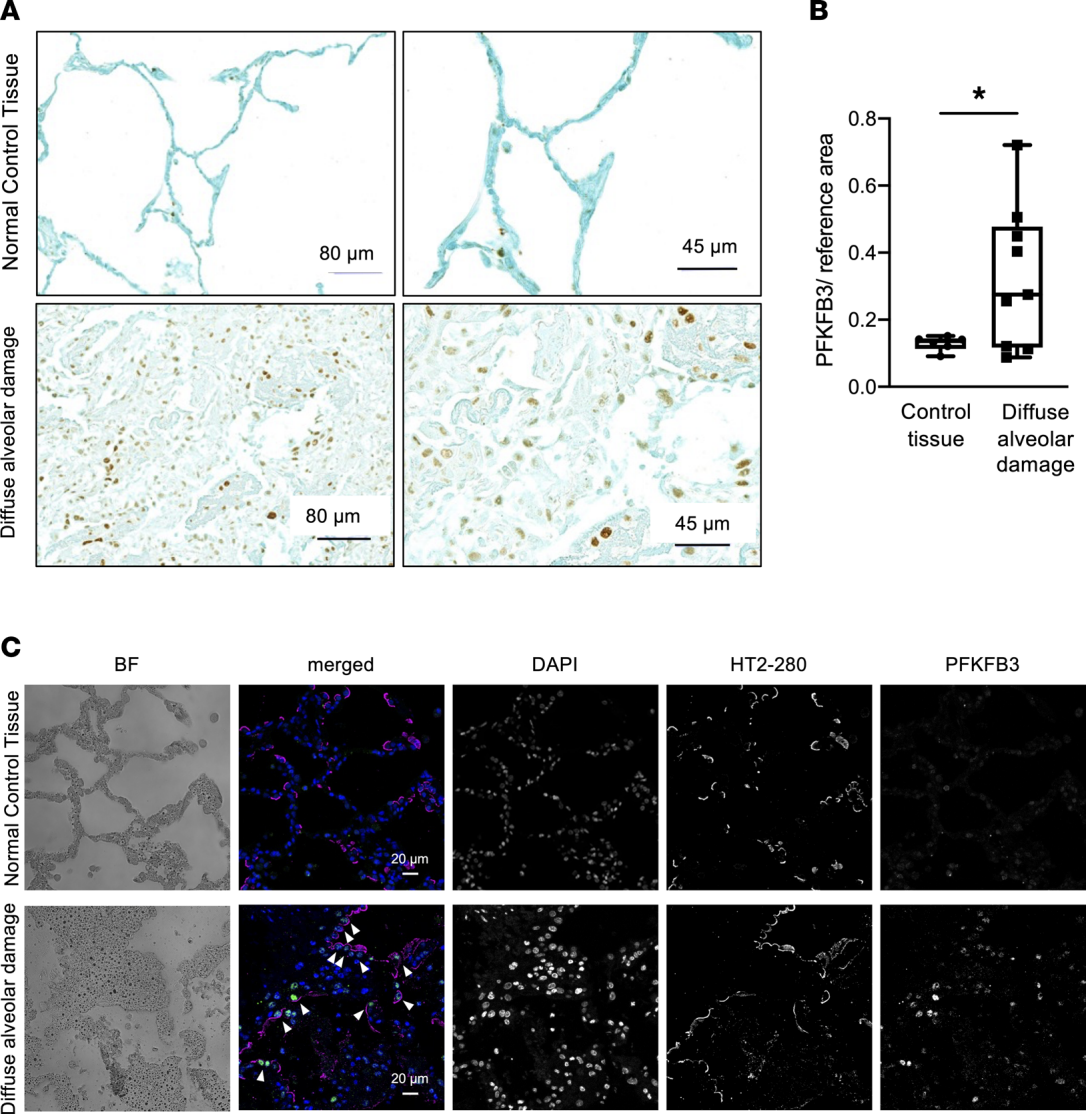
PFKFB3 is apparent in patients with diffuse alveolar damage. (**A**) Representative immunohistochemical staining of lung biopsy tissue specimen from a patient with diffuse alveolar damage (the histologic manifestation of ARDS) and control specimen. (**B**) Quantification of PFKFB3 expression in lung tissue of patients with diffuse alveolar damage (*n* = 9) compared with control patients (*n* = 6). Control specimens were lung biopsies from lungs rejected for transplants. Box-and-whisker plots illustrating the significant difference in mean PFKFB3 expression observed between control and patients with diffuse alveolar damage. For each plot, box bounds represent the first quartile (lower bound) and third quartile (upper bound). Lines within the box represent the median. Whiskers represent the difference from the minimum value observed in the data set to the first quartile (lower whisker) and the difference from the third quartile to the maximum value observed (upper whisker). *P < 0.05. Data were analyzed with 2-tailed, unpaired Student’s *t* test. (**C**) Representative images of lungs from control lungs and patients with diffuse alveolar damage, which were stained with ATII cell marker anti–HT2-280 and anti-Pfkfb3 antibody. The anti–HT2-280 antibody colocalizes with Pfkfb3 within many alveolar epithelial cells (white arrowheads). Images were obtained with 20× objective. All scale bars represent 20 μm. BF, bright-field.

We performed proof-of-principle studies in lung biopsies to address the expression and localization of PFKFB3 during human ARDS. For this purpose, we applied immunohistochemistry staining of human lung biopsy samples that showed diffuse alveolar damage, which represents the histologic manifestation of ARDS. There is a scarcity of lung biopsy specimens from patients with ARDS, which is a major limitation for translational research in ALI. Patients with ARDS rarely undergo lung biopsies, and if a biopsy is obtained, this is frequently due to specific clinical considerations (Supplemental Figure 6). The patient characteristics of lung biopsy samples analyzed have been described previously (40) and are summarized in Supplemental Figure 6. In control samples (lung biopsy samples obtained from lungs that were evaluated for suitability for transplant), expression of PFKFB3 was only minimal (Figure 8). However, in biopsy samples from patients with diffuse alveolar damage, PFKFB3 expression was significantly increased (Figure 8, A and B). Moreover, costaining with ATII marker HT2-280 and PFKFB3 revealed that PFKFB3 colocalized in the ATII cells (Figure 8C and Supplemental Figure 7). In summary, these findings indicate the likelihood that human PFKFB3 expression is elevated during ARDS and is located in ATII cells.

## Discussion

We examined the functional role of alveolar epithelial PFKFB3 and its transcriptional regulation by HIF1A during alveolar inflammation in experimental models of ARDS. Previous studies had suggested that alveolar epithelia, notably ATII cells, increase their ability to use carbohydrate glycolysis during injurious conditions (12). To gain additional insight into the role of alveolar epithelial carbohydrate metabolism during ALI, we initially conducted an unbiased screen in murine ATII cells isolated after exposure to ALI. This screen pointed us toward PFKFB3, which is 1 of the 4 tissue-specific iso-enzymes. Subsequent pharmacologic and genetic in vivo studies demonstrated a protective role of alveolar epithelial PFKFB3 during ALI induced by IMV or acid aspiration. For example, mice with alveolar epithelial *Pfkfb3* deletion (*Pfkfb3^loxP/loxP^* SPC-Cre-ER^+^ mice) failed to elevate their glycolytic response and concomitantly experienced exacerbated lung injury during murine models of ARDS. Studies to address the upstream mechanism of PFKFB3 regulation showed stabilization of alveolar HIF1A protein levels during ALI, leading to elevated PFKFB3 levels. Moreover, reconstitution experiments with i.t. pyruvate given before the onset of lung injury partially rescued the phenotype of *Pfkfb3^loxP/loxP^* SPC-Cre-ER^+^ or *Hif1a^loxP/loxP^* SPC-Cre-ER^+^ mice and attenuated inflammation in acid-induced ALI when given 6 hours after the initiation of ALI. Finally, proof-of-principle studies in lung biopsies of patients with histologic diagnosis of ARDS (diffuse alveolar injury) demonstrated elevated PFKFB3 levels in their lungs, while costaining with the alveolar epithelial markers localized PFKFB3 specifically to alveolar epithelia. In summary, these studies indicate a protective role of ATII specific PFKFB3 by promoting glycolytic responses during ALI and lay the groundwork for ARDS treatment approaches using glycolytic intermediates, such as pyruvate.

In mammals, 4 PFKFB genes (*PFKFB1*, *PFKFB2*, *PFKFB3*, and *PFKFB4*) have been described, which code for the respective iso-enzymes. These isoforms share a highly conserved catalytic core domain (85%) but differ greatly in their kinetic properties and responses to regulatory signals (41). PFKFB1 was initially identified in rat liver and muscle tissue, whereas PFKFB2 isoforms are predominantly expressed in the myocardium (42), while PFKFB4 is predominantly expressed in the testis (43). The PFKFB3 isoform is ubiquitously expressed and has been implicated in glucose metabolism during neoplastic disease (44) but also plays a critical role in endothelial cells, coordinating angiogenic sprouting during physiologic states or malignancy (29). More recently, it has been appreciated that the initially described tissue specificity for PFKFB iso-enzymes is not entirely exclusive and that more than one isoform can be present in one tissue (45). The current findings suggest a potentially novel role for ATII cell PFKFB3 in attenuating alveolar inflammation during ALI. Interestingly, studies on the role of endothelially expressed Pfkfb3 suggest that inducible deletion of Pfkfb3 is protective during pulmonary hypertension (46) or during LPS-induced ALI (47). This underscores the cell type– and injury-specific role of Pfkfb3.

Previous studies have implicated other enzymatic control points for alveolar epithelial carbohydrate metabolism in the regulation of alveolar inflammation during ALI. For example, a study on the role of alveolar carbohydrate metabolism during ALI suggested that the functional activity of succinate dehydrogenase (SDH) is inhibited in response to cyclic mechanical stretch, such as occurs during IMV (12), and implicated mitogen-activated protein kinase activation (48) or increased levels of the competitive SDH inhibitor itaconate (49) in the mechanism of SDH inhibition during ALI (20). SDH has been described to have 4 distinct subunits, SDHA–SDHD, with SDHA carrying the enzymatic activity that catalyzes the conversion of succinate to fumarate (50). Subsequent studies of ALI in mice with inducible alveolar epithelial *Sdha* deletion (*Sdha^loxP/loxP^* SPC-Cre-ER^+^ mice) revealed reduced lung inflammation, improved alveolar barrier function, and attenuated histologic injury, in conjunction with elevated succinate levels (20). Succinate has previously been shown to function as an inhibitor of the prolyl hydroxylases (PHDs) (51) and can thereby function to promote the stabilization of HIF during normoxic conditions. In fact, the protective effects of SDHA inhibition or deletion during ALI, and the concomitant elevations of pulmonary succinate levels, have been linked to succinate-mediated HIF1A stabilization (20). To expand on our previous studies and to further investigate the relationship between succinate and PFKFB3, we tested whether succinate was able to directly trigger PFKFB3 activity. However, as we found that supplementation with cell-permeable succinate did not affect PFKFB3 activity directly, we postulate that succinate stabilizes HIF1A, which, in turn, induces PFKFB3 transcription. Taken together with the current studies, it is conceivable that SDHA inhibition and enhanced PFKFB3 function in coordination to provide lung protection during ALI, with SDHA inhibition and elevation of succinate causing PHD inhibition and HIF stabilization. Increased levels of HIF1A can function to transcriptionally induce PFKFB3, and thereby enhance alveolar epithelial glycolysis, resulting in attenuated alveolar inflammation.

Several previous studies are in line with the current findings for a protective role of the transcription factor HIF in dampening mucosal inflammation during ARDS. Furthermore, mice exposed to lower oxygen concentration had increased HIF1A stabilization and subsequently improved outcomes during polymicrobial sepsis (52). The protective role of HIF1A has since been confirmed in several other models of ARDS, including a highly clinically relevant model of virally induced ALI (12, 25, 53). Of note, clinical trials to investigate the role of HIF activators in COVID-19 patients for prevention or treatment of ARDS are currently underway (ClinicalTrials.gov Identifier: NCT04478071). As shown here, HIF can function to optimize alveolar epithelial glycolysis, though other studies implicate HIF in the regulation of purinergic signaling events (13, 54) or in the induction of microRNAs that can potentially modulate the expression of alveolar epithelial microRNAs (55) that dampen proinflammatory signaling pathways in the alveolar epithelium. HIF1A has been shown to regulate the transcription of PFKFB3 (45). Based on our observation in animals with genetic and pharmacologic inhibition of PFKFB3, we show that PFKFB3 inhibition is sufficient to exacerbate ALI independently from HIF1A. Taken together, these findings indicate a protective role of HIF signaling during alveolar inflammation and indicate that different molecular mechanisms could contribute to this phenomenon.

In the current studies, we used i.t. delivery of pyruvate for reconstitution of a normalized phenotype in *Pfkfb3^loxP/loxP^* SPC-ER-Cre^+^ or *Hif1a^loxP/^* SPC-ER-Cre^+^ mice and found therapeutic effects on the resolution of ALI in wild-type mice. It is important to remember that the functional outcomes of metabolic reconstitution may have cell-specific differences. For example, metabolic intermediates such as succinate have been previously shown to enhance a proinflammatory phenotype in cells of the innate immune system (56), which needs to be considered when using metabolites therapeutically. However, the dual role of glycolytic intermediates that quench alveolar epithelial inflammation while simultaneously enhancing myeloid inflammatory response could likely be beneficial for ARDS treatment. For example, reducing alveolar inflammation can function to dampen pulmonary edema and improve alveolar capillary function, while allowing myeloid cells to perform at a high level during pathogen elimination (55). Pyruvate is metabolized to acetyl-CoA, the entry point into the Krebs cycle by pyruvate dehydrogenase. Pyruvate and its metabolites have been implicated in a hyperoxia-induced model of ALI where a differential function between adult and neonatal mice has been reported (57). Furthermore, antiinflammatory potential with tissue-protective effects of its derivate ethylpyruvate has been demonstrated in several in vivo models of acute inflammation (58). Taken together, these findings suggest a potential therapeutic role for metabolic intermediates such as pyruvate as modulators of inflammation in ARDS, furthering our understanding of lung inflammation and innate protective counterregulatory mechanisms.

Taken together, the present studies identify a selective role for alveolar epithelial PFKFB3 in promoting alveolar integrity through enhancing the capacity for glycolytic carbohydrate metabolism during ARDS. Since alveolar epithelial cells represent the first line of pulmonary defense during ARDS, their vulnerability and the extent of alveolar inflammation are linked directly to ARDS outcomes (10, 59). As such, the current studies highlight that therapeutic strategies (e.g., through HIF activators or via metabolic reconstitution) should be further explored since enhancement of alveolar epithelial glycolysis could represent a new powerful strategy to attenuate lung inflammation during ARDS.

## Methods

### Materials.

Unless otherwise noted, chemicals were obtained from MilliporeSigma. 3PO was obtained from Callbiochem. Fructose-2,6-bisphosphate was obtained from Best of Chemicals Sciences.

### Human lung samples.

Deidentified lung samples with diffuse alveolar damage (that is, the pathologic diagnosis of ARDS) were obtained from the Department of Pathology archives of the University of Colorado. Noninjured controls (donor lungs that were rejected for transplantation) were obtained through the Pulmonary Hypertension Breakthrough Initiative. The patient characteristics of the ALI group have been published previously (40).

### Mice.

Male and female mice were used for the studies and were matched for weight, age, and sex. All animals were housed under a 12-hour light/12-hour dark cycle, and experiments were conducted (age and weight matched) between 10 and 16 weeks of age. Wild-type (C57BL/6), *Hif1a^loxP/loxP^* (B6.129-Hif1a^tm3Rsjo/J^, The Jackson Laboratory stock 007561), and Ubc-Cre (UBC-cre/ERT2, The Jackson Laboratory stock 007001) mice were purchased from The Jackson Laboratory (JAX). Mice with Cre exclusively expressed in ATII cells, SPC-ER-Cre^+^ [Sftpc^tm1(cre/ERT2)Blh^, JAX stock 028054], were provided by Bridget Hogan (Duke University, Durham, North Carolina, USA) (32). *Pfkfb3^loxP/loxP^* (Pfkfb^3tm1Pec^) mice were provided by Katholieke Universiteit Leuven, Leuven, Belgium (29). Whole-body *Hif1a^loxP/loxP^* mice were produced by crossing *Hif1a^loxP/loxP^* mice with Ubc-Cre^+^ mice (35). For tissue-specific knockout in the ATII cells, *Hif1a^loxP/loxP^* and *Pfkfb3^loxP/loxP^* were crossed with SPC-ER-Cre^+^ animals. SPC-ER-Cre^+^ and *Hif1a^loxP/loxP^* mice were described previously (20, 35). Conditional knockout was induced by 75 mg/kg/d tamoxifen over 5 days i.p. as described before (60). Genotyping PCR from tails was used to confirm specific deletion of the floxed area of *Pfkfb3* and *Hif1a*, as well as Cre and flox expression (GeneTyper). Ubc-Cre^+^ animals served as control animals for *Hif1a^loxP/loxP^* Ubc-Cre^+^. SPC-Cre-ER^+^ animals served as controls for the ATII cell–specific knockout animals *Pfkfb3^loxP/loxP^* SPC-Cre-ER^+^ and *Hif1a^loxP/loxP^* SPC-Cre-ER^+^ (of note 1 SPC-ER-Cre control group for IMV and acid aspiration was utilized for Figures 3–7). Control animals also received i.p. tamoxifen. All genetically modified mouse strains had litter sizes and frequencies comparable to wild-type mice. We did not observe an increased frequency of congenital malformation in the genetically modified mice (C57BL/6 background) compared to C57BL/6 wild-type mice.

### Murine models of ALI.

Murine models for acid aspiration and IMV were performed as described previously (61). Briefly, IMV was induced with pressure-controlled ventilation using high inspiratory pressure of 45 mbar for the experimental group and 15 mbar for the control group. FiO_2_ (100%), respiratory rate (80/min), and PEEP (3 mbar) were kept the same in IMV and control groups. Prior to connecting to the ventilator, animals were anesthetized with pentobarbital (70 mg/kg i.p. for induction and 20 mg/kg/h for maintenance). After tracheotomy, a tracheal tube was connected to a mechanical ventilator (Siemens Servo 900C and Draeger Evita 2 Dura, with pediatric tubing). Mice were ventilated for 4 hours or until a cardiac standstill was observed. For acid aspiration, animals were intubated with a 22 G catheter via guide wire using a small animal laryngoscope (Penn-Century), and 50 μL of 0.125 M HCl was instilled. Control animals received 50 μL of 0.9 M NaCl (pH controlled to HCl).

### Isolation of alveolar epithelial cells.

ATII cells were isolated (62). Briefly, mice were deeply anesthetized with 70 mg/kg i.p. pentobarbital. Mice were exsanguinated and lungs were then lavaged with sterile PBS via the right ventricle. Corning dispase (Thermo Fisher Scientific) was then instilled i.t. followed by a low–melting point agarose plug. En bloc–removed lungs were incubated for 45 minutes at room temperature. Tissue was teased apart and passed through a 70 μm strainer (BD Biosciences). The cell mixture was then labeled with a mixture of anti-CD16/32 (catalog 553143), anti-TER119 (catalog 553672), anti-CD45 (catalog 3553078), and anti-CD90 (catalog 554896) (all from BD Biosciences) and subsequently incubated with streptavidin labeled with magnetic beads (Promega) to negatively select for ATII cells. As a final step, fibroblasts were removed by adherence to a Petri dish for 2 hours. To control the purity of the isolated cells, cells were stained for EpCAM expression with immunofluorescence microscopy.

### Metabolite analysis of alveolar epithelial cells.

Metabolites from frozen cell pellets were extracted using ice-cold methanol/acetonitrile/water (5:3:2) at a ratio of 2^6^ cells/mL by vortexing 30 minutes at 4°C. Samples were clarified through centrifugation (10 minutes at 400*g*, 4°C), and 10 μL of supernatant was analyzed using a 5-minute C18 gradient on a Thermo Fisher Scientific Vanquish UHPLC coupled online to a Thermo Fisher Scientific Q Exactive mass spectrometer operating in positive and negative ion modes (separate runs) as previously described in detail (63). Samples were normalized to cell count.

### Cell culture and treatment.

MLE-12 cells (obtained from ATCC, CRL-2110) and primary ATII cells were cultured as described previously (64, 65) in DMEM with 4.5 g/L glucose and stable l-glutamine, 10% FBS, and 1% penicillin/streptomycin mix (all from Corning Cellgro). Cells were incubated in a humidified atmosphere of 5% CO_2_/95% air at 37°C.

### In vitro stretch model.

To recapitulate cyclic mechanical stretch, we utilized a previously described in vitro model (12) resembling mechanical ventilation. Briefly, cells were plated on BioFlex culture plates (Flexcell) that were coated with collagen type 1 (MLE-12 cells) or fibronectin (primary isolated ATII cells), and cells were allowed to attach. MLE-12 cells were grown to 80% confluence; ATII cells were seeded with a density of 3 × 10^6^/well (6-well plate). All cells were cultured in 4 mL medium (DMEM, 4.5 g/L glucose, 10% FBS, 0.02% l-glutamine). Plates were then placed on a Flexcell FX_4000T Tension Plus System. Cells were stretched at 30% and sine wave 5 seconds on, 5 seconds off. Cells were then collected at specified time points and processed for further analysis. Control cells were cultured under identical conditions without application of cyclic mechanical stretch.

### PFKFB3 activity.

PFKFB3 activity was assessed by measuring intracellular fructose-2,6-bisphosphate (F2,6BP) levels as previously described (28, 66). In brief, cell pellets were obtained by centrifugation at 376*g* for 10 minutes at 4°C. The pellets were resuspended in 50 mM Tris acetate (pH 8.0) and 100 mM NaOH, incubated at 80°C for 5 minutes, and placed on ice. Cell lysates were neutralized to pH 7.2 with 1 M acetic acid and 1 M HEPES and then incubated at 25°C for 2 minutes in 50 mM Tris, 2 mM Mg^2+^, 1 mM fructose-6-phosphate, 0.15 mM NAD, 10 U/L pyrophosphate-dependent PFK-1, 0.45 kU/L aldolase, 5 kU/L triosephosphate isomerase, and 1.7 kU/L GAPDH. Pyrophosphate (0.5 mM) was added and the rate of change in absorbance (OD = 339 nm) per minute over 5 minutes was determined with a Thermo Fisher Scientific GENESIS 40-Vis spectrophotometer. A calibration curve using 0.1 to 1 pmol of F2,6BP was used to calculate F2,6BP, which was then normalized to total protein.

### Sample collection.

Lungs were lavaged 3 times (each time 1 mL of PBS) to obtain BALF. Retrieved lavage fluid was then centrifuged at 300*g* for 5 minutes at 4°C, and resulting cell-free BALF was immediately snap-frozen for subsequent measurement of protein and ELISA studies. For pulmonary tissues, the lungs were flushed with 10 mL saline via the right ventricle and either snap-frozen in liquid nitrogen and stored at −80°C or conserved in formalin for histologic analysis.

### Measurement of BALF protein content and cytokine concentrations.

Protein content of BALF was measured via Bradford Assay as described previously (67). For measurements of cytokines via ELISA, BALF samples were thawed to 4°C, and specific cytokine concentrations (IL-1β, IL-6, KC/GRO [which is the CXCL1 equivalent], and TNF-α) in BALF were determined using a V-PLEX Proinflammatory multiplex ELISA panel (Meso Scale Diagnostics). Cytokine concentrations were normalized to BALF protein content.

### Wet/dry ratio and arterial oxygen partial pressure measurement.

Animals were euthanized, 100 μL blood was taken from the aorta, and arterial blood gas was measured with an i-STAT1 blood gas analyzer (Abbott Laboratories). Wet/dry ratio of lungs was obtained as previously described (68).

### Cell differential in BALF.

Cell differential was performed by transferring undiluted BALF onto slides (Cytospin, Thermo Fisher Scientific). Slides were then stained with Hema 3 Stat Pack (Thermo Fisher Scientific). Ten random images were obtained at 20× original magnification. Neutrophils and monocytes/macrophages were counted manually, with a blinded protocol.

### Measurement of pyruvate in BALF and whole-lung tissue.

Pyruvate was measured in whole-lung tissue and BALF with a commercially available colorimetric assay (MilliporeSigma) according to the manufacturer’s instructions. Briefly, enzyme mix and dye reagent were added to BALF or homogenized whole-lung tissue, and absorbance was measured at 579 nm.

### Targeted carbohydrate metabolism mRNA screen.

Mouse RT^2^ Profiler PCR Array Glucose Metabolism (PAMM-006Z, QIAGEN) was used to screen a panel of 84 genes involved in glucose metabolism in ATII cells. Total RNA was isolated and quantified using a NanoDrop 2000 (Gene Company Limited), and cDNA was generated using RT^2^ First Strand Kit (QIAGEN). cDNA was mixed with 2× RT^2^ SYBR Green qPCR Master Mix (QIAGEN) and double-distilled H_2_O. The qPCR was performed on a Bio-Rad iCycler according to the RT^2^ Profiler PCR Array instructions under these PCR running parameters: 95°C for 10 minutes, then 40 cycles at 95°C for 15 seconds and 60°C for 1 minute. Each array contained 5 separate housekeeping genes (β-actin [Actb], B2m, Gapdh, Gusb, and Hsp90ab1); we utilized Actb for normalization of the sample data. Array data were normalized against the housekeeping genes by calculating the ΔCt for each gene of interest in the plate. Fold changes of gene expression were analyzed and generated by using RT^2^ PCR array data analysis web portal version 3.5. Genes that had fold changes of more than 2 in expression against the control group were considered significant. The candidate genes were validated by individual qPCR. Array data were deposited in the NCBI’s Gene Expression Omnibus (accession GSE220132). Supplemental Table 1 contains lists of genes and their respective *P* values.

### Transcriptional analysis (qPCR).

Total RNA was isolated from primary alveolar epithelial cells, MLE-12 cells, or murine lung tissue using QIAGEN RNeasy Mini Kit by following manufacturer’s protocol, and cDNA was generated using iScript cDNA Synthesis Kit (Bio-Rad). RNA transcript levels were determined by real-time reverse transcription PCR (iCycler, Bio-Rad). Primers were obtained from Quantitect (QIAGEN): mouse Actb (QT01136772), IL-6 (QT00098875), CXCL1 (QT00115647), PFKFB1 (QT00285096), PFKFB2 (QT00154000), PFKFB3 (QT00109284), PFKFB4 (QT00136738), GAPDH (QT00097146), PFK (QT00155911), ENO1 (QT00260442), LDHA (QT02325414), and HK3 (QT01561252).

### Immunoblotting.

Western immunoblotting was used to measure HIF1A or PFKFB3 protein content. For Western immunoblotting, cells were washed twice with ice-cold PBS and lysed with 1× cell lysis buffer (Cell Signaling Technology) including protease inhibitor PMSF (effective concentration 100 μg/mL). For immunoblotting of HIF1A, nuclear protein fraction was extracted from alveolar epithelial cells using an NE-PER Nuclear and Cytoplasmic Extraction Reagents Complete Protease Inhibitor (Thermo Fisher Scientific). Protein concentrations were determined using Quick Start Bradford dye reagent (Bio-Rad), and equal protein amounts were denatured in sample buffer, separated by SDS-PAGE, transferred to nitrocellulose membranes, blocked in 5% skim-milk (*w/v*) in TBS including 1% Tween-20 (TBST) (v/v), and then probed with the respective primary antibodies at 4°C overnight. After incubation with primary antibodies, membranes were washed with TBST 3 times for 10 minutes and then incubated with secondary HRP anti-rabbit (Cell Signaling Technology catalog 7074) or HRP anti-mouse (Cell Signaling Technology catalog 7076) for 1 hour. Proteins were detected by ECL (Thermo Fisher Scientific). Nuclear loading control was Lamin A/C and cytoplasmatic loading control was β-actin. Densitometry was performed with ImageJ (NIH). Primary antibodies were PFKFB3 (catalog 181861, Abcam), HIF-1 alpha (catalog NB100-105 clone H1alpha67, Novus Biologicals, Bio-Techne), β-actin (catalog A5441, MilliporeSigma), and Lamin A/C (catalog sc-4777, Santa Cruz Biotechnology).

### Histopathological evaluation of ALI.

Lungs were explanted and prepared for paraffin embedding as described (61). We stained 5 μm sections with H&E. Assessment of histological lung injury was performed by grading as follows (61): infiltration or aggregation of inflammatory cells in airspace or vessel wall: 1 = only wall, 2 = few cells (1 to 5 cells) in airspace, 3 = intermediate, 4 = severe (airspace congested); interstitial congestion and hyaline membrane formation: 1 = normal lung, 2 = moderate (<25% of lung section), 3 = intermediate (25%–50% of lung section), 4 = severe (>50% of lung section); and hemorrhage: 0 = absent, 1 = present. Six representative images were obtained by from each animal and were analyzed by 2 investigators with a protocol blinded to group assignments.

### Immunohistochemistry staining.

Detection of PFKFB3 by immunohistochemistry in human and mouse lungs was performed after deparaffinization and antigen retrieval with citrate solution (pH 6.0; 30 minutes) as described previously (69). Slides were incubated with primary antibody against PFKFB3 (catalog 181861; 1:300; 1 hour, Abcam), followed by treatment with secondary Dako EnVision rabbit system K 4010. Sections were then counterstained with Light Green (StatLab Medical Products). Slides were then coded, and images of the lung parenchyma were captured in an unbiased fashion, using the 40× objective. The intensity of expression was then quantified on coded images, using a macro developed for Metamorph software (Molecular Devices), which integrates intensity (in pixels) and area (in pixels) of the positive immunohistochemistry staining, generating arbitrary units. Following decoding, data were analyzed by comparison among experimental groups.

### Immunofluorescence staining.

Sections of lungs from patients with ARDS and control lungs were stained with both the anti-PFKFB3 antibody (catalog 181861, Abcam) and an anti–HT2-280 antibody (catalog TB-27AHT2-280, Terrace Biotech) to identify ATII cells (39). The sections were treated with Antigen Unmasking Solution, Citrate-Based H-3300-250 (Vector Laboratories), blocked with a mixture of 10% donkey serum and 10% goat serum, and reconstitutedin antibody diluent for 1 hour at room temperature. The slides were incubated with a combination of anti-PFKFB3 (1:100) and anti–HT2-280 antibody (1:200) or rat IgG (negative control, Vector Laboratories, catalog I-4000-1) at a dilution of 1:200 applied for 1 hour at room temperature. A combination of secondary antibodies, Alexa Fluor 488 donkey anti-mouse (catalog A-21202, Invitrogen, Thermo Fisher Scientific) and Alexa Fluor 555 goat anti-rabbit (catalog A-21428, Invitrogen, Thermo Fisher Scientific), each diluted 1:200, was applied for 1 hour along with DAPI. Sections were then treated with TrueVIEW Autofluorescence Quenching Kit (catalog SP-8400-15, Vector Laboratories). Images from the double-stained slides were examined under a Zeiss LSM900 confocal microscope. Images were obtained utilizing ZEN 3.1 (Zeiss) software.

### Statistics.

We assessed the severity of ALI by determining mRNA expression levels of proinflammatory cytokines, pulmonary barrier function, protein content in the BALF, and histology. For analysis of alveolar epithelial cells, each data point was isolated from a different animal. For cell line experiments, each experiment was carried out independently. For analysis of histology, 2 investigators, following a protocol blinded to genotype and treatment, performed scoring independently. All statistical analyses were carried out using GraphPad Prism 8.1.2 (GraphPad Software). We conducted Grubbs’ tests on all eligible data to examine outliers. No outliers were identified. All data approximately followed normal distributions and were summarized as means ± SD. For comparison between 2 groups, 2-tailed Student’s *t* test was used. In cases of comparison between more than 2 groups, 1-way ANOVA with Tukey’s correction for multiple comparisons was applied. The Mantel-Cox test was used for analysis of survival curves. *P* values less than 0.05 were considered statistically significant. The authors had full access to the data and have read and agree to the manuscript as written.

### Study approval.

For mice, the local institutional animal care and use committee (University of Colorado, Anschutz Medical Campus) approved all animal experiments: protocol numbers B104914(06)1D, B104917(04)E, and 00128. Animal experiments were carried out in accordance with the US Law on the Protection of Animals and the NIH *Guide for the Care and Use of Laboratory Animals* (National Academies Press, 2011).

For humans, lung samples with diffuse alveolar damage diagnosis were obtained through the pathology archives of the University of Colorado. The deidentified human lung control samples were obtained through the Pulmonary Hypertension Breakthrough Initiative Research Network. The Colorado Multiple Institutional Review Board, Aurora, Colorado, USA, approved all human protocols and waived the requirement for informed consent for the use of archived, deidentified, paraffin-embedded lung samples.

## Author contributions

CUV, RMT, and HKE designed the research studies. CUV, EC, CS, EKV, and NB conducted the experiments. CUV, NB, and XY analyzed the data. PC provided knockout animals. RMT and EPS provided access to patient samples. All original research data were reviewed by CUV, EC, NB, XY, RMT, and HKE. CUV wrote the manuscript and provided all figures. RMT, ESN, XY, KS, and HKE revised the manuscript.

## Supplementary Material

Supplemental data

## Version 1 11/03/2022

In-Press Preview

## Version 2 12/22/2022

Electronic publication
