# Supplementary material for: HIF1A-dependent induction of alveolar epithelial PFKFB3 dampens acute lung injury
Source: JCI Insight. 2022 Dec 22;7(24):e157855. doi: 10.1172/jci.insight.157855 (PMC9869967; doi:10.1172/jci.insight.157855)

Supplementary Figure 1

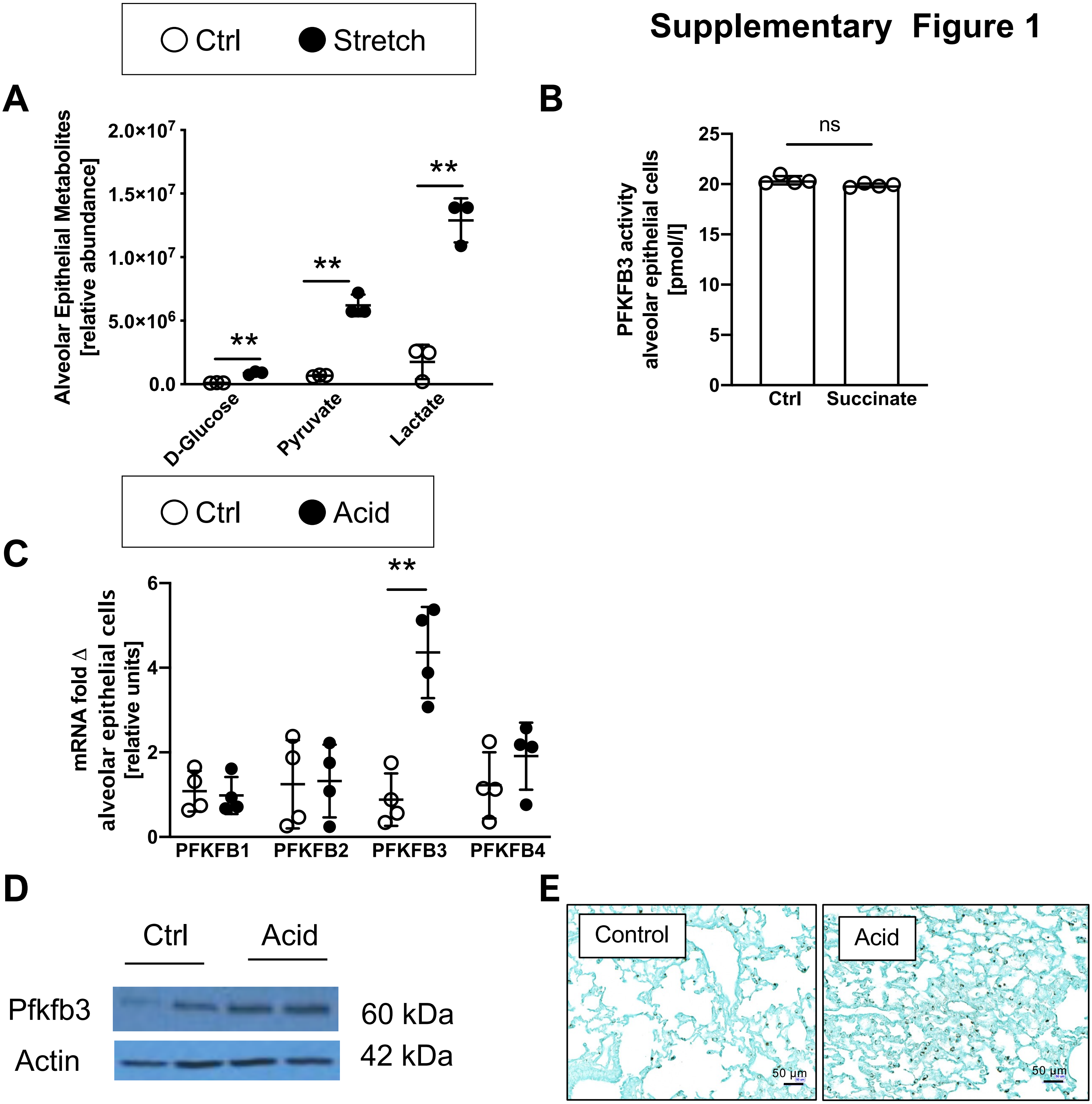

S1: Alveolar epithelial cells upregulated glycolysis in response to cyclic mechanical stress and Pfkfb3 is upregulated in response to acid induced ALI *in vivo*

(a) alveolar epithelial cells were subjected to cyclic mechanical stretch for 24 hours *in vitro* and their glycolytic intermediates were determined by mass spectrometry, n=3. Measurement of PFKFB3 activity in alveolar epithelial MLE-12 cell line *in vitro, n=4*. (c-e) C57BL/6 mice were given 50 µl of 0.1 M HCl to simulate acid aspiration, control animals received 50 µl of pH controlled NaCl. After 3 days lungs were harvested. (c) PFKFB isoform mRNA expression was determined via qPCR in alveolar epithelial cells, n=4. (d) PFKFB3 protein expression in alveolar epithelial cells was determined by Western blot. (e) Immunohistochemical expression of PFKFB3 in mouse lungs after 3 days in control (i.t. NaCl) and acid (i.t. HCl) instillation. Panel C: 4 male and 4 female. Panel D, E: 2 male and 2 female. Data are represented as mean ± SD, n 3-4, ** p< 0.01, ****p<0.0001. Data were analysed with two-tailed, unpaired, student's t-test

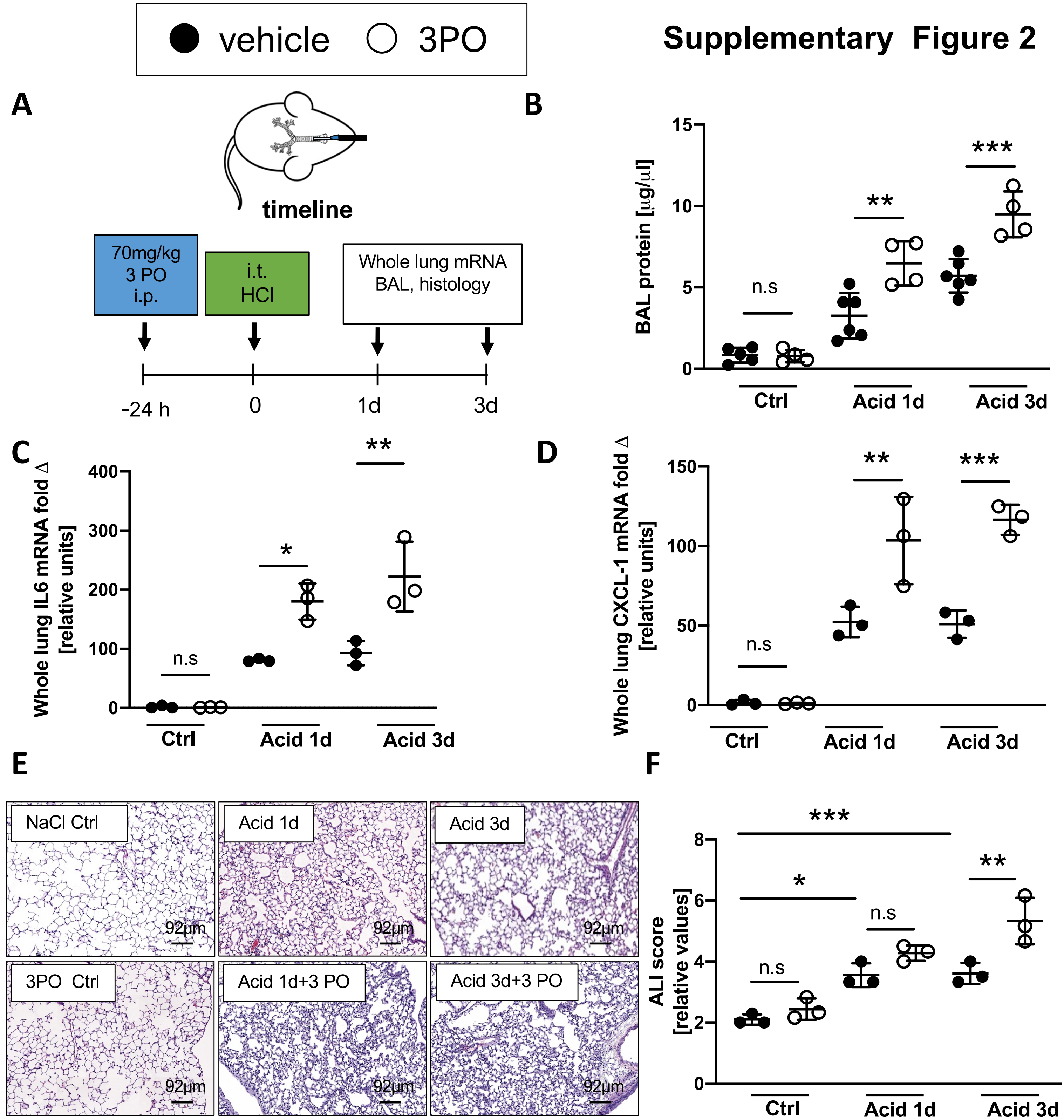

S2: Pharmacological inhibition of PFKFB3 exacerbates acid aspiration

(a) Schematic of the experiment: 8-10-week-old C57BL/6 mice received 70mg/kg of PFKFB3 inhibitor 3PO i.p 24 hours prior to induction of ALI with acid instillation with i.t. HCl. Control groups received vehicle. After 1 and 3 days the lungs were removed. (b) Protein concentration measured in BALF with Bradford-Assay, n=5/4/6/4/5/4. (c, d) IL-6 and CXCL-1 mRNA expression in whole lung tissue was determined by qPCR, n=3/group. (e, f) Representative images of H&E stained lungs from mice subjected to acid aspiration, with controls having received pH-controlled NaCl (e) and cumulative lung injury score which is a a combined score of cellular infiltrates, interstitial congestion and hyaline membrane formation and hemorrhage (f), n=3/group. Panel B: 14 male and 14 female. Panel C-F: 9 male and 9 female Data are represented as mean ± SD, n.s not significant ,* $p< 0.05$, ** $p< 0.01$, ***$p<0.001$. Data were analysed with two-tailed, unpaired, student's t-test.

Supplementary Figure 3

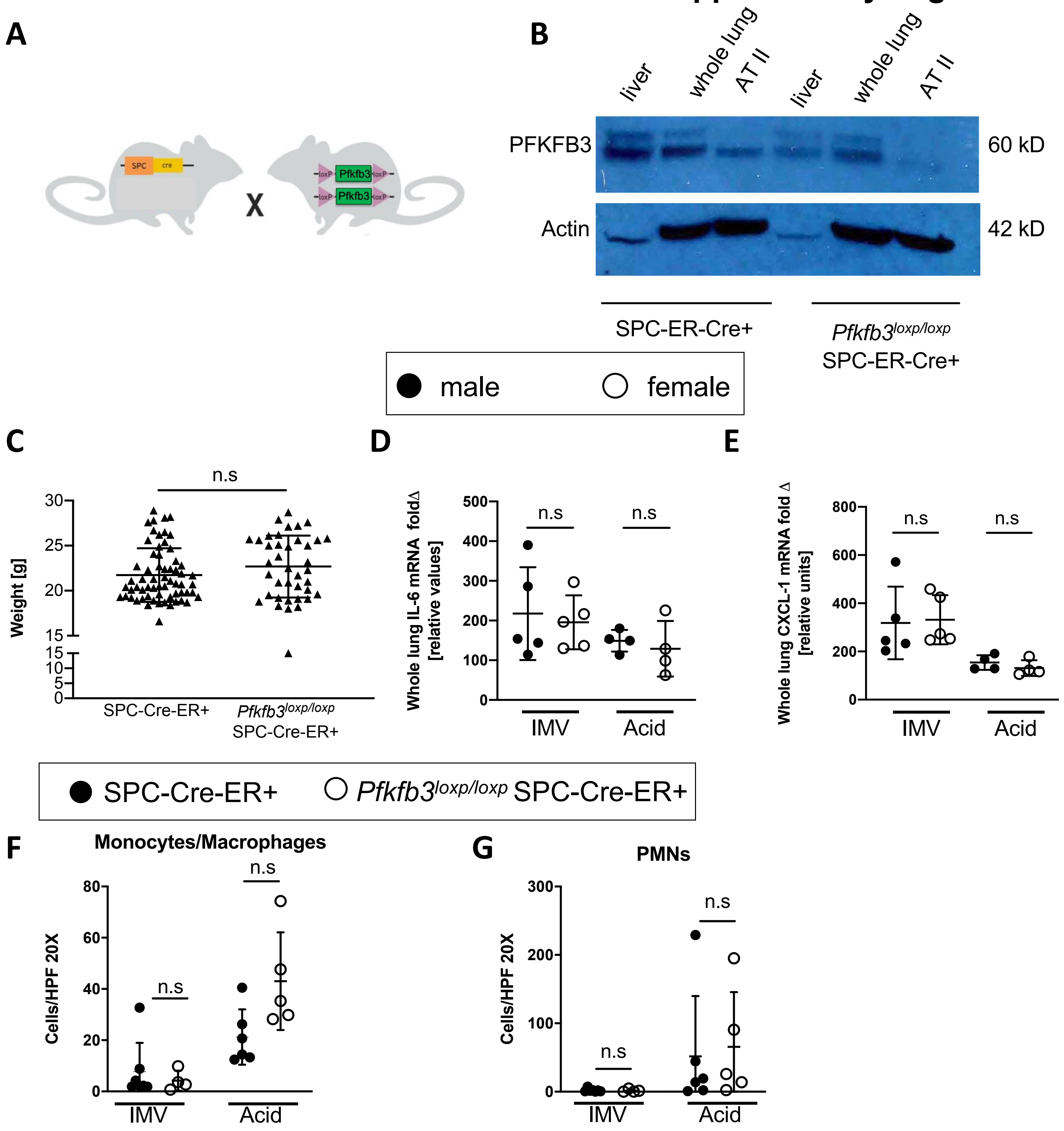

S3: Characterization of mice with alveolar epithelial specific deletion of *Pfkfb3*
(a) Model depicting breeding scheme of transgenic *Pfkfb3*$^{loxp/loxp}$ SPC-Cre-ER+ mouse line. (b) Western Blot analysis of PFKFB3 expression in alveolar epithelial cells, whole lung and liver tissue from *Pfkfb3*$^{loxp/loxp}$ SPC-Cre-ER+ and SPC-Cre-ER+ control animals (2 male, 2 female). (c) Weights of *Pfkfb3*$^{loxp/loxp}$ SPC-Cre-ER+ 10-12 weeks after tamoxifen induction. *Pfkfb3*$^{loxp/loxp}$ SPC-Cre-ER+ n=39 with 20 female and 19 male animals and with and SPC-Cre-ER+ n=63 animals with 31 female and 32 male animals. (d-e) mRNA expression of cytokines was determined in whole lung tissue with qPCR (8 male,10 female), n=5/5/4/4. (f, g) BALF was obtained from mice after induction and differential cell count was obtained (n= 7/4/4/5)(10 male, 10 female). Data are represented as mean ± SD, n.s not significant. Data were analyzed with two-tailed, unpaired, student's t-test

Supplementary Figure 4

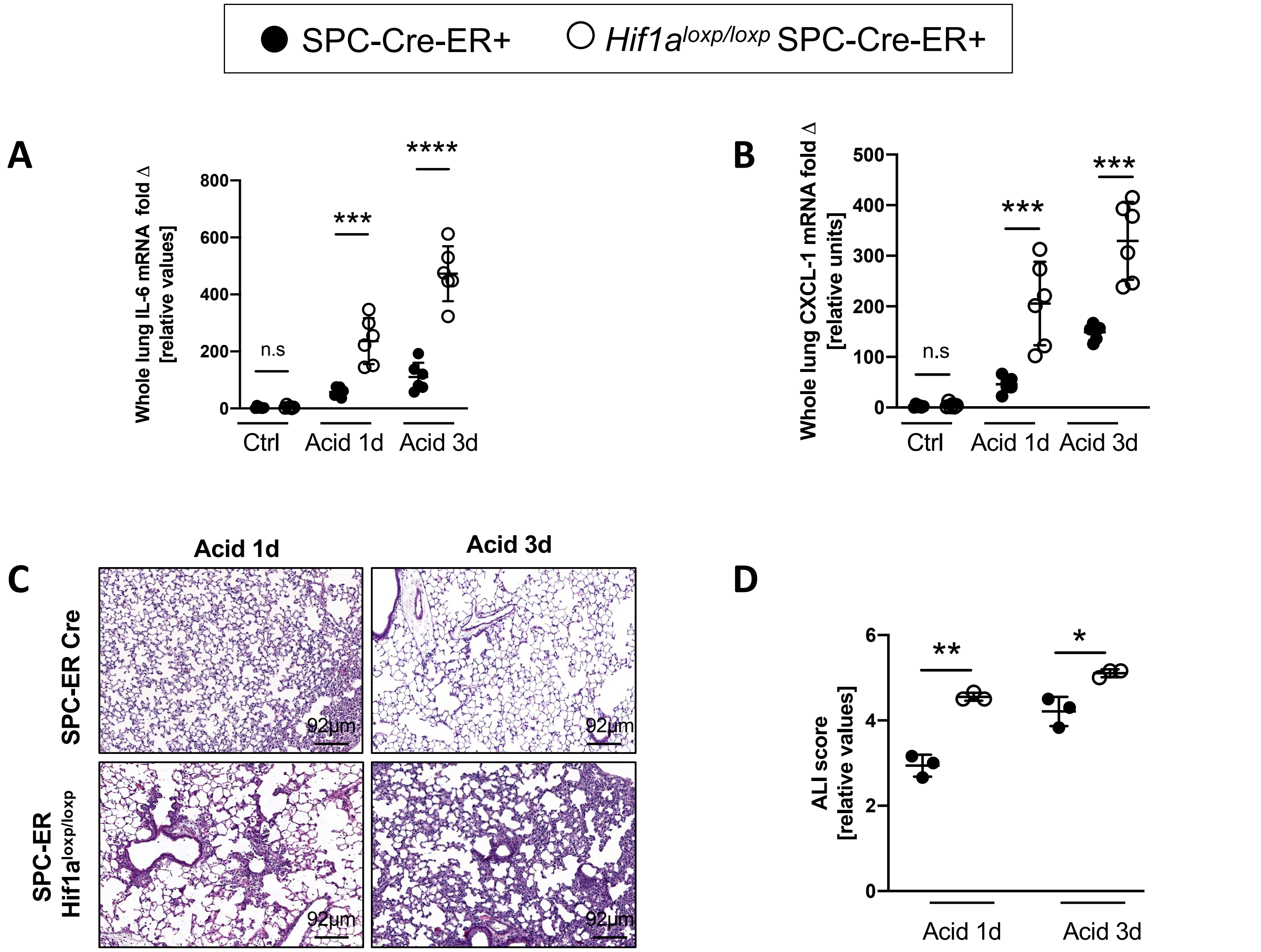

S4: Alveolar epithelial HIF1A is protective in acid aspiration induced ALI

(a-d) *Hif1a*$^{loxp/loxp}$ Surfactant Cre+ (*Hif1a*$^{loxp/loxp}$ SPC-Cre-ER+) mice or age, sex, and weight-matched controls (SPC-ER-Cre+) were exposed to acid instillation to induce lung injury. (a, b) IL-6 and CXCL-1 mRNA expression in whole lung tissue was determined by qPCR (n=6/group) (18 male, 18 female). (c, d) Representative images of H&E stained lungs from mice subjected to acid induced acute lung injury (c) and cumulative lung injury score (d) which is a combined score of cellular infiltrates, interstitial congestion, and hyaline membrane formation and hemorrhage (n=3/group) (7 males, 5 female). Data are represented as mean ± SD, n.s not significant, * p< 0.05, **p<0.01,***p<0.001, ****p<0.0001. Data were analyzed with two-tailed, unpaired, student's t-test

Supplementary Figure 5

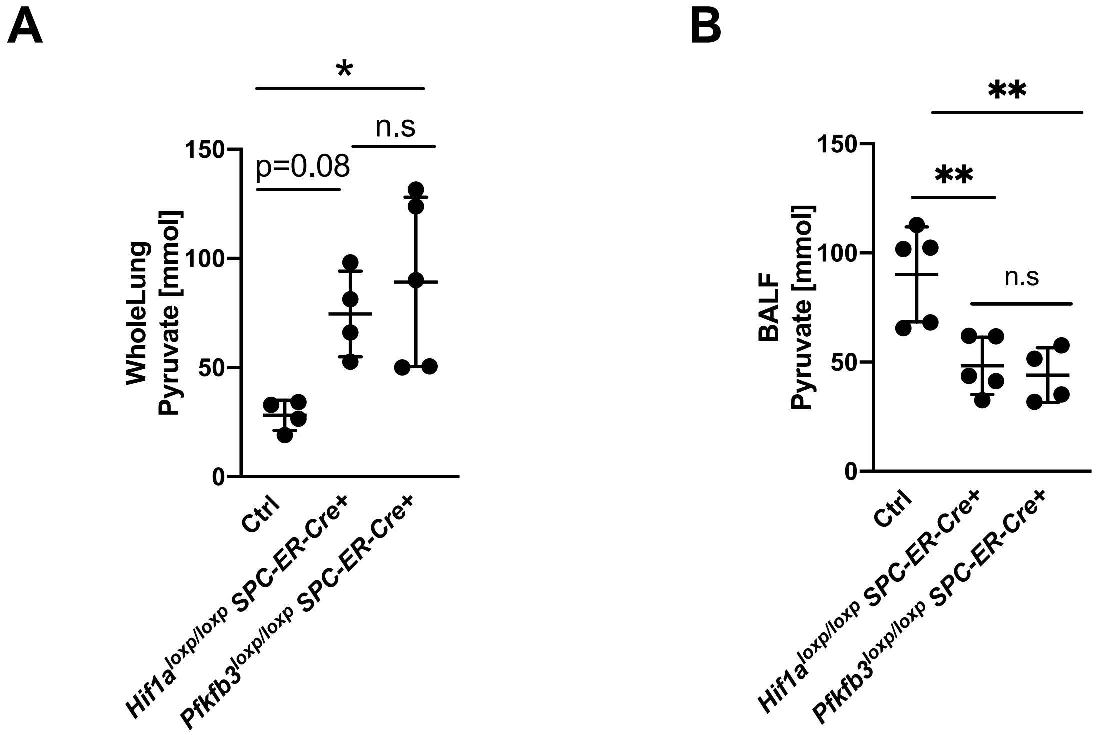

S5: Measurement of pyruvate levels in whole lung homogenate and BALF after i.t. pyruvate instillation
10-15 week old mice matched weight and sex were used in all experiments. Alveolar epithelial cell specific conditional knockout mice (Panel A : 6 males, 7 females, Panel B: 7 males, 7 females). *Hif1a* $^{loxp/loxp}$ SPC-ER-Cre+ and *Pfkfb3*$^{loxp/loxp}$ SPC-ER-Cre+ or control animals (SPC-ER-Cre+) received 200mg/kg i.t. pyruvate 15 min prior to induction of IMV. After 4 hours lung tissue and BALF were harvested for analysis. Pyruvate was measured in whole lung homogenate (A) n=4/4/5 or BALF (B) n=5/5/4 with a commercially available calorimetric assay. Data are represented as mean ± SD. * $p < 0.05$,** $p<0.01$, n.s. =not significant. Data were analyzed with 1-way ANOVA with Tukey's correction for multiple comparisons.

Supplementary Figure 6

**Patient demographics**	**Clinical history**	**Pathologic diagnosis** (from surgical lung biopsy)	**Lung PFKFB3 IHC intensity** (fold increase from mean IHC intensity in normal lungs n=6)
63 y/o female	Remote history of solid organ transplantation. Septic shock and respiratory failure	Acute and organizing diffuse alveolar damage	3.46
44 y/o male	Remote history of solid organ transplantation. Sepsis and respiratory failure	Diffuse alveolar damage with focal organizing pneumonia	0.67
48 y/o male	Chemotherapy-induced respiratory failure	Acute lung injury	3.9
55 y/o female	Acute exacerbation of interstitial lung disease	Acute lung injury with diffuse alveolar damage and focal organizing pneumonia	0.87
45 y/o female	Amiodarone-associated acute respiratory failure	Diffuse alveolar damage, organizing phase	2.12
62 y/o male	Acute exacerbation of interstitial lung disease.	Diffuse alveolar damage with focal alveolar hemorrhage, mild organizing pneumonia and focal interstitial fibrosis	5.56
57 y/o male	Recent organ transplantation. Septic shock with acute respiratory failure	Acute and organizing diffuse alveolar damage	0.93
51 y/o male	Remote history of solid organ transplantation. Acute respiratory failure	Severe diffuse alveolar damage	1.97
40 y/o male	Unknown	Pneumocystis jirovecii infection with diffuse alveolar damage.	3.11

S6: Patient characteristics of samples with diffuse alveolar damage

Demographic, clinical, and pathologic information from patients with lung biopsies demonstrating diffuse alveolar damage (the histologic correlate of ARDS). The samples obtained from the Pathology Archives of the University of Colorado were published before (32). We calculated the fold intensity increase of the PFKFB3 staining of each samples compared to the mean PFKFB3 intensity staining of the 6 control lungs.

Supplementary Figure 7

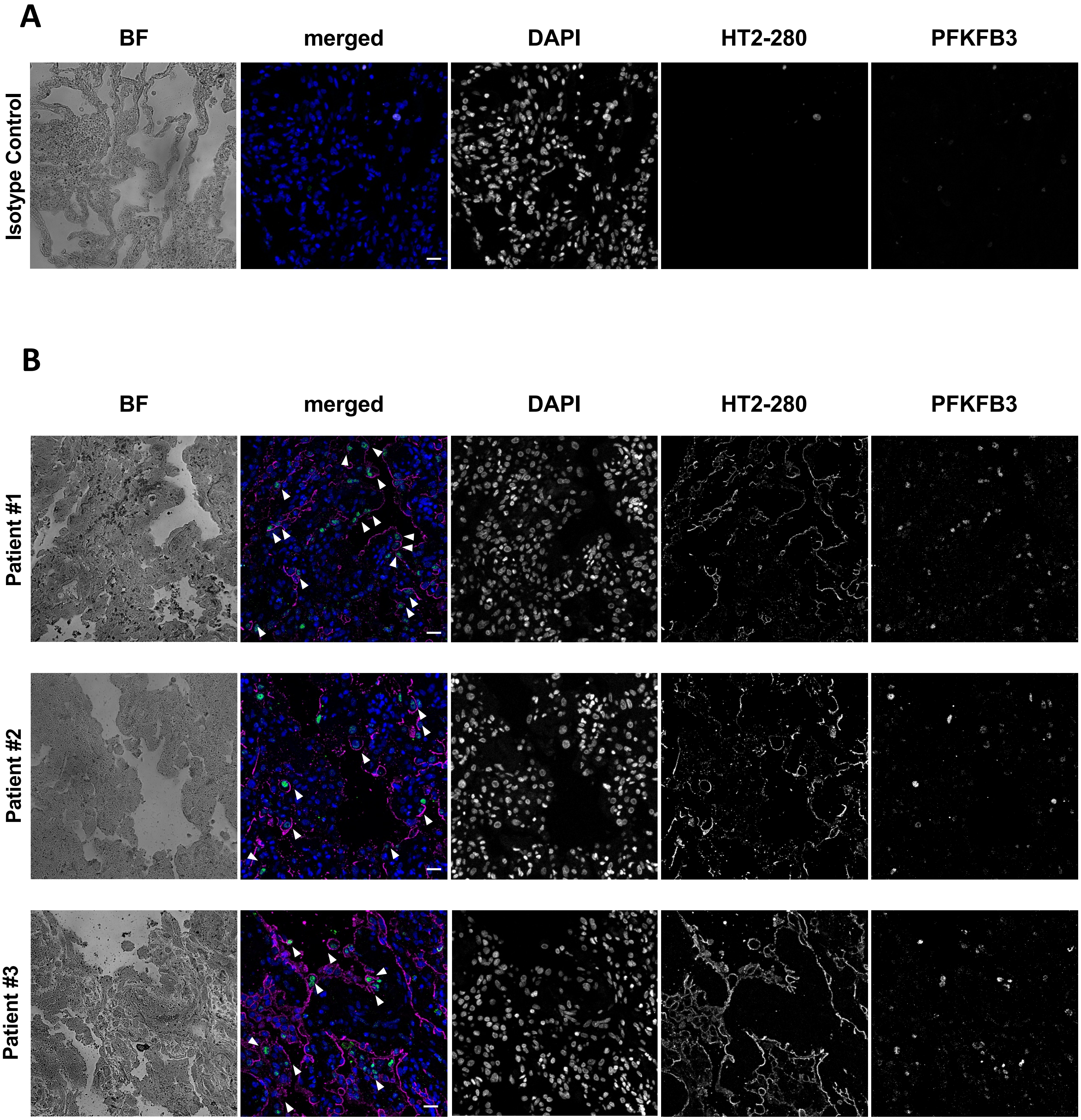

S7: Immunofluorescence staining demonstrates colocalization of PFKFB3 in alveolar epithelial cells in patients with the diagnosis of diffuse alveolar damage in lung biopsies. . Co-staining with isotype controls does not demonstrate significant co-localization (A).Representative images of lungs, that were stained with AT II cell marker HT2-280 and anti-PFKFB3 antibody in human lung biopsies from patients with diffuse alveolar damage the histologic equivalent of ARDS(B). The anti-HT2-280 antibody colocalizes with PFKFB3 within many alveolar epithelial cells (white arrowheads). Images were obtained with 20x objective. BF= bright field.. All scale bars are 20 µm.

Supplementary Table 1

p-values

Gene symbol	Ub-Cre+	Hif1a loxp/loxp-Ub-Cre
Acly	0.750491	0.685866
Aco1	0.834504	0.851446
Aco2	0.303733	0.39169
Agl	0.808715	0.024895
Aldoa	0.81705	0.569922
Aldob	0.444808	0.573756
Aldoc	0.380196	0.290385
Bpgm	0.050802	0.2939
Cs	0.767392	0.098801
Dlat	0.987126	0.286129
Dld	0.563679	0.515758
Dlst	0.980721	0.253007
Eno1	0.044671	0.444761
Eno2	0.880619	0.251949
Eno3	0.721182	0.034305
Fbp1	0.829731	0.834585
Fbp2	0.077898	0.866176
Fh1	0.434739	0.097835
G6pc	0.947446	0.603023
G6pc3	0.561206	0.268302
G6pdx	0.545315	0.539085
Galm	0.269487	0.141286
Gapdhs	0.870906	0.339968
Gbe1	0.153982	0.110818
Gck	0.059714	0.055972
Gpi1	0.716625	0.289946
Gsk3a	0.65205	0.397831
Gsk3b	0.586315	0.876957
Gys1	0.069664	0.080554
Gys2	0.05482	0.160555
H6pd	0.452766	0.354762
Hk2	0.536148	0.675793
Hk3	0.031164	0.812665
Idh1	0.395289	0.040017
Idh2	0.169215	0.265896
Idh3a	0.735415	0.709404
Idh3b	0.607307	0.457323
Idh3g	0.035648	0.063808
Mdh1	0.552596	0.372779
Mdh1b	0.363052	0.77475
Mdh2	0.469409	0.913792
Ogdh	0.047769	0.865339

Gene symbol	Ub-Cre+	Hif1a loxp/loxp-Ub-Cre
Pck1	0.885501	0.752775
Pck2	0.811587	0.870563
Pcx	0.320088	0.558085
Pdha1	0.879239	0.316184
Pdhb	0.68226	0.49919
Pdk1	0.056872	0.169195
Pdk2	0.05726	0.151421
Pdk3	0.608887	0.032597
Pdk4	0.99304	0.625394
Pdp2	0.746609	0.828714
Pdpr	0.868283	0.308381
Pfkl	0.018793	0.06102
Pgam2	0.581784	0.618194
Pgk1	0.94994	0.978486
Pgk2	0.947903	0.60357
Pgm1	0.919478	0.641326
Pgm2	0.200208	0.246792
Pgm3	0.412342	0.745503
Phka1	0.444022	0.782143
Phkb	0.289272	0.34629
Phkg1	0.650799	0.432347
Phkg2	0.718573	0.445922
Pklr	0.272858	0.228031
Prps1	0.052843	0.613468
Prps1l1	0.019031	0.669107
Prps2	0.12812	0.122868
Pygl	0.703574	0.336034
Pygm	0.795961	0.819215
Rbks	0.945927	0.977389
Rpe	0.841696	0.192545
Rpia	0.669123	0.56625
Sdha	0.048976	0.06824
Sdhb	0.842267	0.74737
Sdhc	0.979662	0.015478
Sdhd	0.814984	0.850023
Sucla2	0.610141	0.499166
Suclg1	0.802084	0.776385
Suclg2	0.857056	0.001112
Taldo1	0.768223	0.943355
Tkt	0.710723	0.953889
Tpi1	0.580069	0.477692
Ugp2	0.367632	0.214678

PCR Array: Glucose Metabolism:

Glucose metabolism:

Glycolysis: Aldoa, Aldob, Aldoc, Gpgm, Eno1, Eno2, Eno3, alm, Gapdhs, GckGlp1,Hk2, Hk3, Pfk, Pgam2,Pgk1, Pgk2, Pgm1, Pgm2, Pgm3, Pklr, Tpi1

Gluconeogenesis: Fbp1, Fbp2, G6pc, G6pc3, Pck2, Pox

Regulation:Pdp2, Pdpr, Pdk1, Pdk2, Pdk3, Pdk4

TCA cycle: Acly, Aco1, Aco2, Cs, Dlat, Dld, Dlst, Fh1, Idh1, Idh2, Idh3a, Idh3b, Idh3g, Mdh1, Mdh1b, Mdh2, Ogdh, Pck1,Pck2, Pcx, Pdha1, Pdhb, Sdha, Sdhb,Sdhc, Sdhd, Sucla2, Suclg1, Suclg2

Pentose Phosphate Pathway: G6pdx, H6pd, Prps1, Prps1/1, Prps2, Rbks, Rpe, Rpia, Taldo1, Tkt

Glycogen Metabolism: *Synthesis*: Gbe1, Gys1, Gys2, Ugp2. *Degradation*: Agl,Pgm1, Pgm2,Pgm3, Pygl, Pygm. *Regulation*: Gsk3a, Gsk3b, Phka1, Phkb, Phkg1, Phkg2

p-values for Ub-Cre+ animals were calculated by comparing Ub-Cre+ animals exposed to IMV to control animals. p-values for Hif1aloxp/loxp Ub-Cre+ animals were calculated by comparing Hif1aloxp/loxp Ub-Cre+ with Ub-Cre+ animals (both after IMV exposure). Students's t-test was used. n=4/group

Full unedited gel for Figure 1f

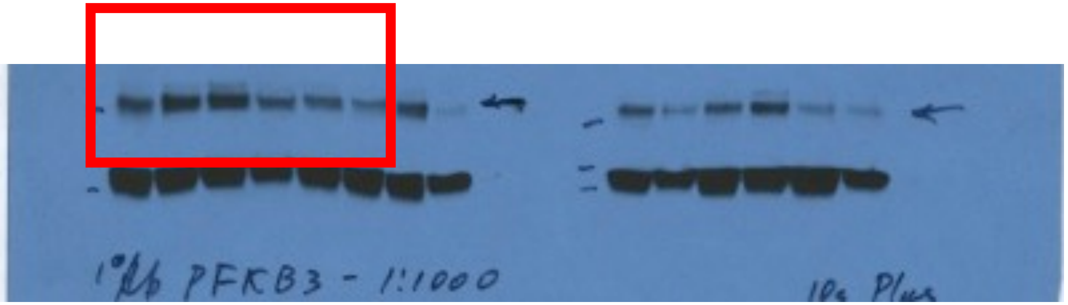

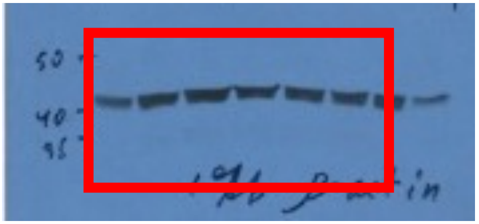

Full unedited gel for Figure 5a

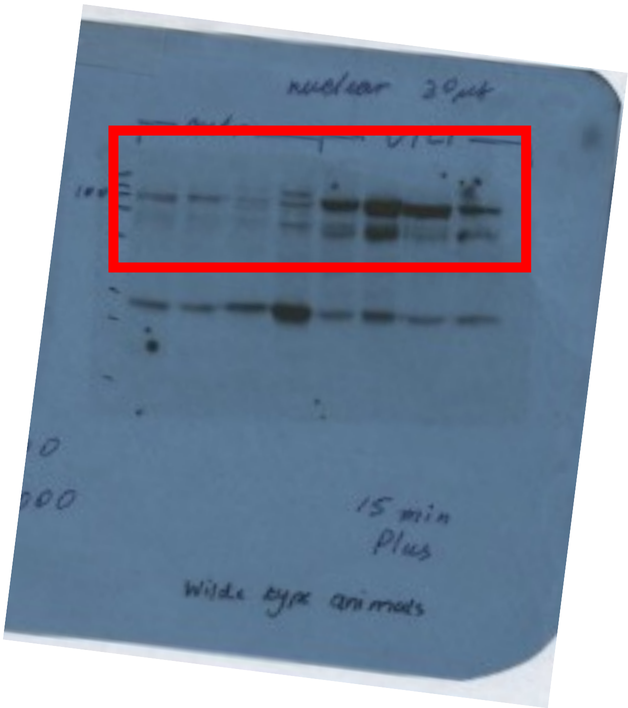

HIF1A

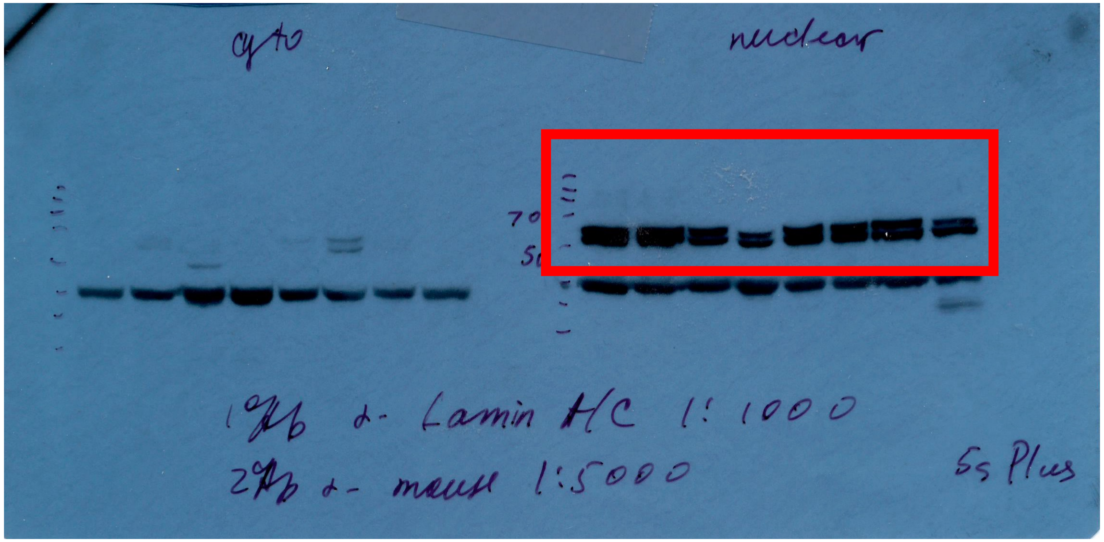

Lamin A/C

Full unedited gel for Figure 5b

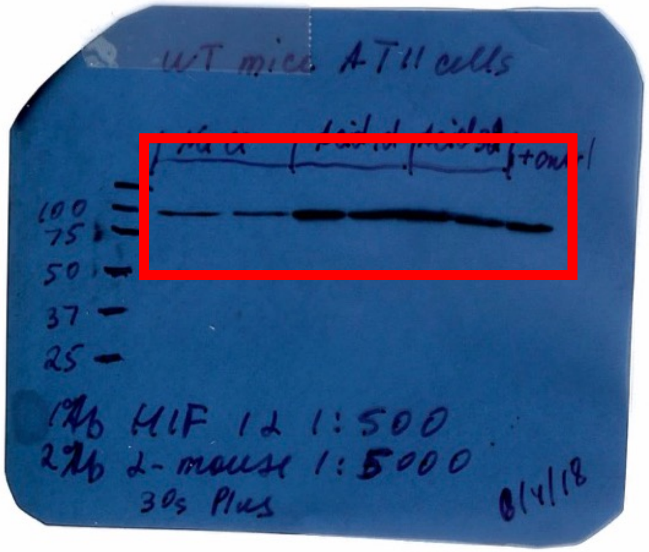

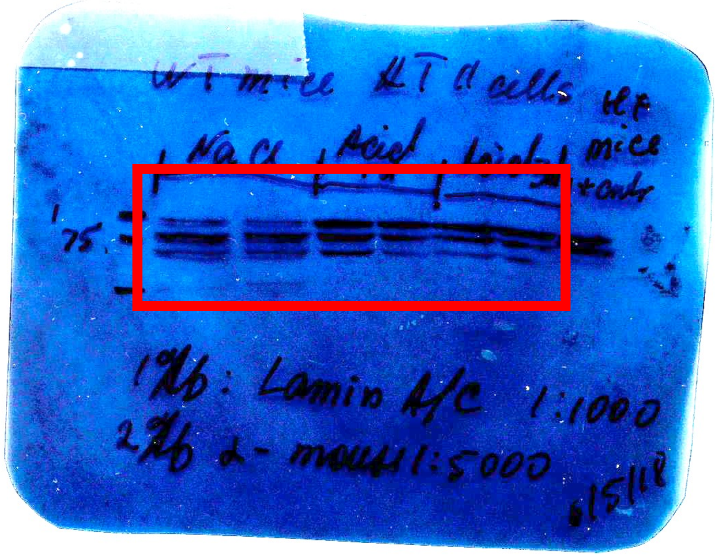

Full unedited gel for Figure S1A

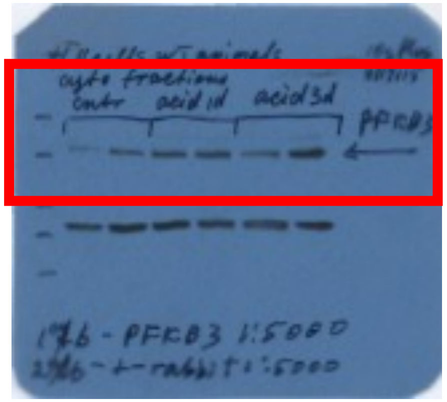

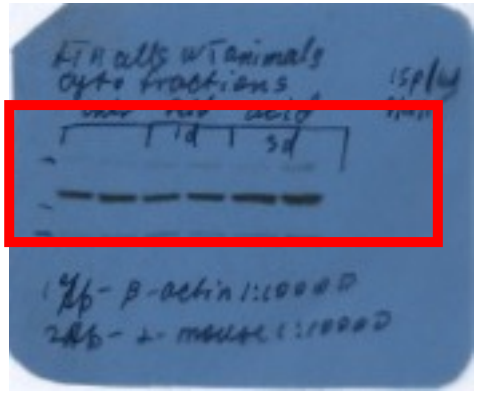

Full unedited gel for Figure S3B

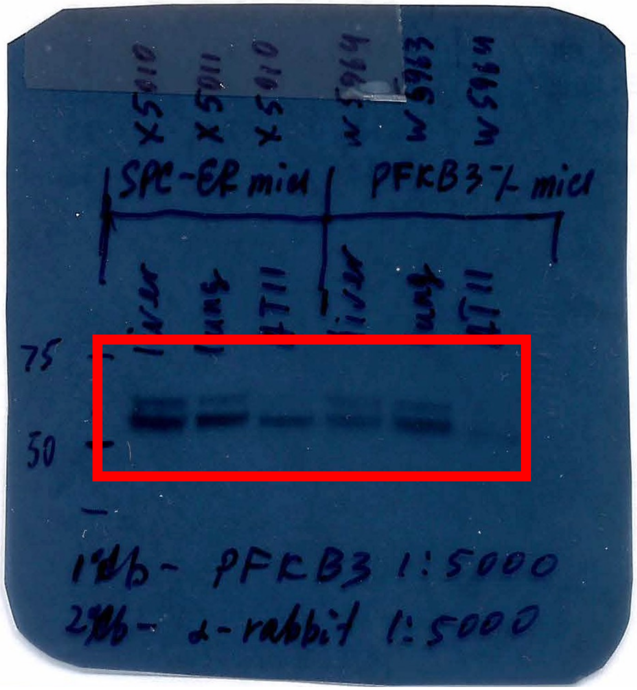

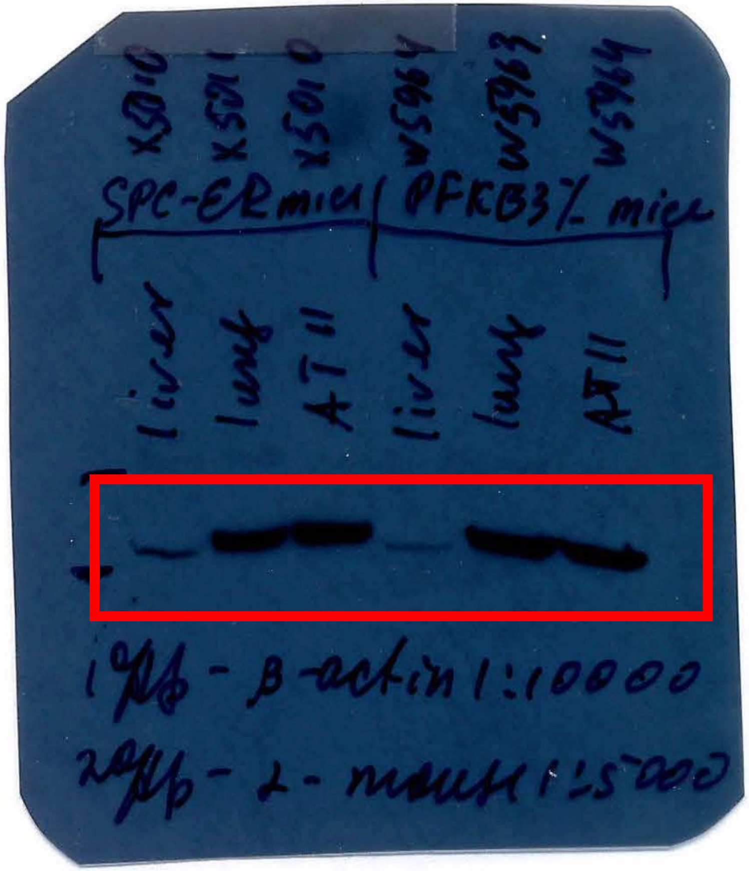

Supplement: Supplemental data [file jciinsight-7-157855-s131.pdf]
